# Insights into Salinity Stress-Induced Morpho-Physiological and Molecular Responses and Nanoparticle- and Nanobiochar-Mediated Tolerance Mechanisms During Seed Germination

**DOI:** 10.3390/nano16150948

**Published:** 2026-07-31

**Authors:** Abhishek Singh, Rupesh Kumar Singh, Mirela Alina Sandu, Veronica Ivanescu, Omkar Singh, Anuj Saraswat, Karen Ghazaryan

**Affiliations:** 1 Applied Ecology and Environmental Research Laboratory, Research Institute of Biology, Faculty of Biology, Yerevan State University, Yerevan 0025, Armenia or intmsc.abhi@gmail.com (A.S.); kghazaryan@ysu.am (K.G.); 2 Centre for the Research and Technology of Agro-Environmental and Biological Sciences (CITAB), Universidade de Trás-os-Montes e Alto Douro (UTAD), Inov4Agro, Quinta de Prados, 5000-801 Vila Real, Portugal; rupesh@utad.pt; 3 Faculty of Land Reclamation and Environmental Engineering, University of Agronomic Sciences and Veterinary Medicine of Bucharest, 59 Marasti, Blvd., District 1, 011464 Bucharest, Romania; veronica.ivanescu@fifim.ro; 4 ICAR-Krishi Vigyan Kendra, P.G. College, Ghazipur 233001, Uttar Pradesh, India; omkar.singh39734@gmail.com; 5 Department of Integrative Agriculture, College of Agriculture and Veterinary Medicine, United Arab Emirates University, Al Ain P.O. Box 15551, United Arab Emirates; anujsaraswattt@gmail.com

**Keywords:** salinity stress, seed germination, nanoparticles, nanobiochar, nanopriming, aquaporins, oxidative stress, germination indices

## Abstract

Soil salinity is a major environmental constraint that threatens global food security by significantly inhibiting seed germination and early seedling establishment. Salinity disrupts all three phases of seed germination: Phase I (imbibition), where reduced water absorption capacity reduces seed hydration and delays metabolic reactivation; Phase II (lag phase), where ionic toxicity and oxidative stress impair enzyme activity, reserve mobilization, and cellular metabolism; and Phase III (radicle protrusion), where limited cell division and length prevent radicle emergence and seedling establishment. These disturbances reduce germination percentage, germination rate, germination index, germination energy, and plant vigor, while increasing average germination time. At the morpho-physiological level, salinity impairs water absorption, membrane stability, photosynthetic pigment accumulation, and root–shoot development. Biochemically, excessive accumulation of reactive oxygen species (ROS), hydrogen peroxide (H_2_O_2_), and malondialdehyde (MDA) causes cellular damage and metabolic dysfunction. At the molecular level, salinity alters the expression of the aquaporin gene family (PIPs, TIPs, NIPs, and SIPs), suppresses starch mobilization by reducing α-amylase, enhances abscisic acid (ABA) signaling, and inhibits gibberellic acid (GA) biosynthesis, all of which cause inhibition of germination and early growth. As a result, an effective strategy is needed to improve seed germination under saline conditions. Therefore, the second focus of this review is to critically evaluate the potential of nanoparticles (NPs) and nanobiochar (NBC) as emerging tools to mitigate salinity stress during seed germination. Current evidence suggests that NPs and NBC enhance water absorption, maintain membrane strength, improve nutrient availability, promote antioxidant defense systems, and regulate osmotic adjustment in saline environments. Furthermore, these nanomaterials alter key molecular pathways involved in aquaporin expression, hormonal homeostasis, and reserve mobilization, thereby promoting successful germination and seedling establishment. By combining recent advances in physiological, biochemical, and molecular research, this review provides a comprehensive understanding of salinity-induced germination disruption and highlights the potential of NP- and NBC-based approaches to improve crop establishment under saline conditions.

## 1. Introduction

**Figure 1:**
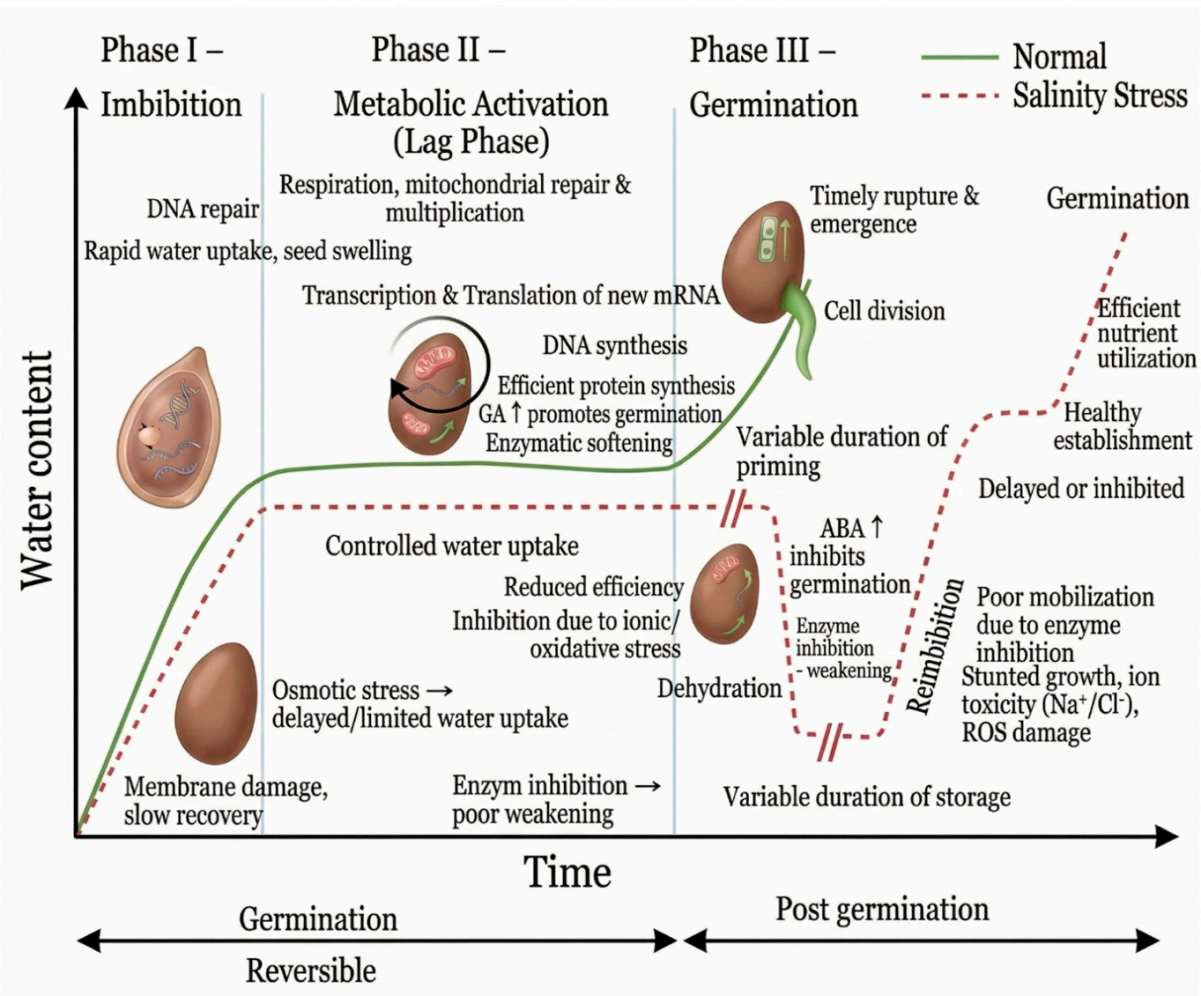
Overview of the impact of salinity stress on the three phases of seed germination. Salinity acts through a distinct dominant modality in each phase: osmotic stress during imbibition (Phase I), where an elevated external osmotic potential reverses the water-potential gradient across the seed coat and restricts water uptake; ionic stress during the lag phase (Phase II), where Na^+^ and Cl^−^ influx produces cytoplasmic ion imbalance, an elevated Na^+^/K^+^ ratio, and suppression of α-amylase activity; and oxidative stress during radicle protrusion and early seedling growth (Phase III), where ROS overproduction drives lipid peroxidation, protein denaturation, and mitochondrial dysfunction. These modalities converge on delayed and reduced germination, poor seedling establishment and diminished crop productivity.

Environmental stress significantly constrains plant growth, crop productivity, and agricultural sustainability [1]. Soil salinity is a major limiting factor for sustainable agriculture, currently affecting approximately 33% of irrigated agricultural land, with projections suggesting that this figure may exceed 50% by 2050 [2]. Salt stress affects more than 6% of the total land area globally, with over 70 countries identified as highly salinized [3]. By restricting vegetative development, delaying fertilization, and impairing seed reproduction, salinity is considered among the most critical abiotic stresses affecting agricultural productivity in semi-arid and arid regions [4]. Salinity stress reduces crop production primarily through osmotic and ionic stress, which impair physiological, biochemical, and molecular functions [5]. Among the various developmental stages affected, seed germination and early seedling establishment are particularly sensitive to salinity-induced water limitation, as salt stress delays germination onset, reduces germination rate and uniformity, and ultimately compromises crop performance and yield [6,7]. Consequently, these early developmental stages are considered key reference points when defining plant salt tolerance, as illustrated in Figure 1.

Salinity stress adversely affects germination and growth parameters across a wide range of plant species [8,9,10]. Increasing salinity reduces osmotic potential, thereby limiting water uptake, and simultaneously induces ion toxicity and oxidative stress [11]. To counteract oxidative damage, plants deploy enzymatic antioxidants—including catalase, ascorbate peroxidase, and superoxide dismutase—as well as non-enzymatic compounds such as carotenoids, proline, α-tocopherol, and ascorbic acid [12]. Seed viability and germination performance are commonly assessed through a set of key indices—germination percentage (GP%), germination rate (GR), germination index (GI), mean daily germination (MDG), mean germination time (MGT), and germination energy (GE)—all of which are negatively affected by salinity stress [13,14,15,16,17,18,19,20,21,22,23,24,25]. Conversely, biochar applied in combination with organic fertilizers has been shown to significantly improve germination rate and plant growth parameters, including plant height and biomass, in vegetable crops [26].

In agriculture, nanotechnology has emerged as a promising approach for improving plant stress tolerance and supporting sustainable farming. Nanoparticles (NPs; 1–100 nm) have demonstrated potential in mitigating the adverse effects of abiotic stresses, including salinity, by improving nutrient uptake, enhancing antioxidant defense, and promoting plant adaptations [27,28]. Nanobiochar (NBC) is a novel carbon-based nanostructured material derived from biochar (BC)—itself produced by pyrolyzing biomass under limited-oxygen conditions at 300–700 °C from a wide range of feedstocks including forest debris, agricultural residues, and vegetable waste [29,30]—and further modified through chemical treatment, ball milling, or gaseous activation to yield particles of nanoscale dimensions (10–100 nm) [28]. Compared to conventional BC, NBC exhibits a larger surface area, increased microporosity, enhanced ion-exchange capacity, and improved adsorption efficiency, all of which contribute to its superior priming effectiveness [28]. Moreover, the physicochemical characteristics of NBC can be tailored through selection of feedstock materials, modification methods, and processing conditions, enabling targeted physiological outcomes [31]. Collectively, NPs and NBC influence key hydrolytic enzymes—including α-amylase, protease, and acid phosphatase—thereby accelerating seed germination and seedling establishment under stress conditions [32,33,34,35].

To understand the impact of salinity stress on seed germination and the potential of nanotechnology-based mitigation strategies in greater depth, this review combines a systematic literature review with a bibliometric analysis. The review is organized into two main sections. The first section provides an in-depth examination of the morphological, physiological, biochemical, and molecular mechanisms by which salinity stress disrupts the three phases of seed germination (imbibition, metabolic activation, and radical protrusion), emphasizing osmotic stress, ionic toxicity, oxidative damage, hormonal imbalance, and associated changes in germination traits, water relations, antioxidant metabolism, and gene regulation. The second section evaluates the role of NPs and NBC in mitigating salinity-induced germination disruptions, and highlights their effects on water absorption, ion homeostasis, antioxidant defense, osmotic adjustment, hormonal regulation, aquaporin expression, reserve mobilization, and stress-responsive pathways.

## 2. Systematic-Review-Based Evaluation of the Effects of Salinity Stress and NPs/NBC on Seed Germination

### 2.1. Methodology

#### 2.1.1. Database and Source

The main data source in this study was the Institute for Scientific Information (ISI) Web of Science (WoS) core databases. WoS is an extensive database covering bibliometric-analysis-relevant fields and providing global access to scientific works across nearly all disciplines, including the natural sciences, biomedicine, and engineering technology. As of early 2026, the Web of Science (WoS) Core Collection indexes over 22,000 journals across indices including SCIE, SSCI, AHCI, and ESCI, with major impact in various research fields [36,37].

#### 2.1.2. Data Selection Parameters

WoS is a research analysis tool. In the literature, the WoS Core Collections sub-database was selected via a topic search (TS) [38]. In this study, we analyzed the effects of salinity stress on seed germination; morphological, physiological, biochemical, and molecular-level traits; and the application of NP- and NBC-mediated tolerance with the following research questions that directed this study:

(1) What are the current trends in the publication, production, and citation of scientific articles related to seed germination and NPs-based tolerance strategies?
(2) Which countries are most involved in research related to seed germination and NPs-based tolerance strategies?
(3) What are the most relevant keywords related to seed germination and NPs-based tolerance strategies that can help identify future trends and research gaps?

**Figure 2:**
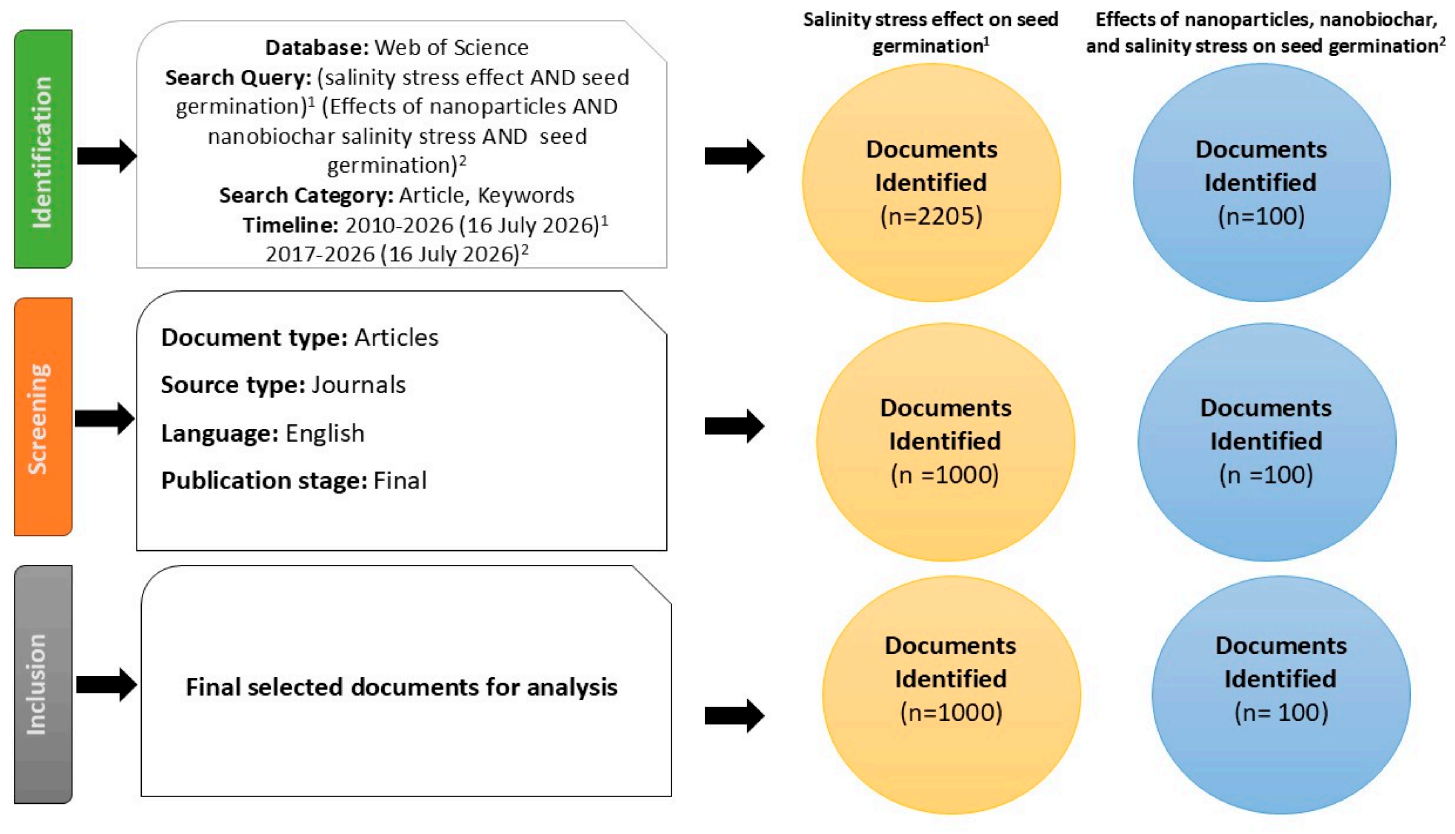
PRISMA 2020 flow diagram of the study identification, screening, and inclusion process (final = 1000) related to studies that focus on salinity stress and seed germination ^1^ and studies that focus on NP- and NBC-based salinity stress mitigation for the promotion of seed germination ^2^. Note: ^1^ and ^2^ show search query keywords and description.

PRISMA (Preferred Reporting Items for Systematic Reviews and Meta-Analyses) was used to identify and analyze studies on salinity stress and its impact on seed germination traits from 2010 to 2026 (up to 16 July 2026). Additionally, NPs and NBC (2017–2026, up to 16 July 2026) were included to mitigate salinity stress and enhance seed germination [39]. Data collection and selection included identification, screening, and eligibility determination with information extraction (Figure 2) [40]. The search formula was TS (*Salinity Stress Effect AND Seed Germination*) (*Effects of Nanoparticles AND Nanobiochar Salinity Stress AND Seed Germination*).

#### 2.1.3. Tools for Data Extraction and Bibliometric Analysis

In this study, RStudio version 4.5.3 (11 March 2026 ucrt), served as the bibliometric analysis tool for evaluating performance and creating scientific maps. RStudio is widely used for handling and analyzing bibliographic data, facilitating the extraction of details on documents, authors, sources, and research themes [41].

### 2.2. Results and Critical Discussion

**Figure 3:**
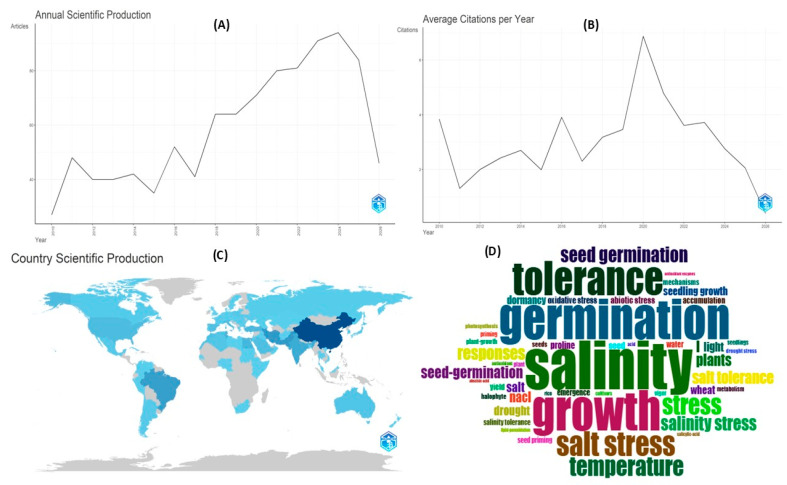
Bibliometric overview of (**A**) scientific publications, (**B**) citations, and (**C**) global research by country publication distribution and (**D**) a word cloud of keywords on salinity stress and seed germination from 2010 to 2026.

The annual scientific output of publications related to salinity stress and seed germination is depicted in Figure 3A, which shows the trend over time in the number of studies published on this topic. The results show that research output has gradually increased since 2010, with a significant increase after 2018 and peak productivity in 2023–2024, reflecting growing scientific interest in understanding and mitigating salinity-related disruptions to plant germination. The citation trends for published articles show fluctuations over the years, with the highest average citation impact observed around 2020, reflecting the publication of highly influential studies during this period (Figure 3B). The subsequent decline in recent years is likely due to the fact that newly published articles have accumulated fewer citations. Geographical analysis highlights global research engagement in salinity and seed germination studies. China emerged as the largest contributor, followed by India, the United States, and several European and Asian countries, reflecting the global importance of salinity stress as a major agricultural challenge (Figure 3C). Darker shades indicate greater publication output.

The word cloud in Figure 3D shows the most frequently occurring keywords in the analyzed literature. Keywords such as salinity, germination, growth, tolerance, salt stress, and stress indicate that research is primarily focused on understanding the effects of salinity on seed germination and plant growth, as well as the mechanisms underlying salt tolerance and stress adaptation [13,14]. The size of each word is proportional to its frequency of occurrence in the dataset. Overall, the bibliometric analysis reveals a rapidly growing research field focused on the effects of salinity on seed germination and plant development, with increasing global participation and an emphasis on stress-tolerance mechanisms and sustainable crop-improvement strategies.

**Figure 4:**
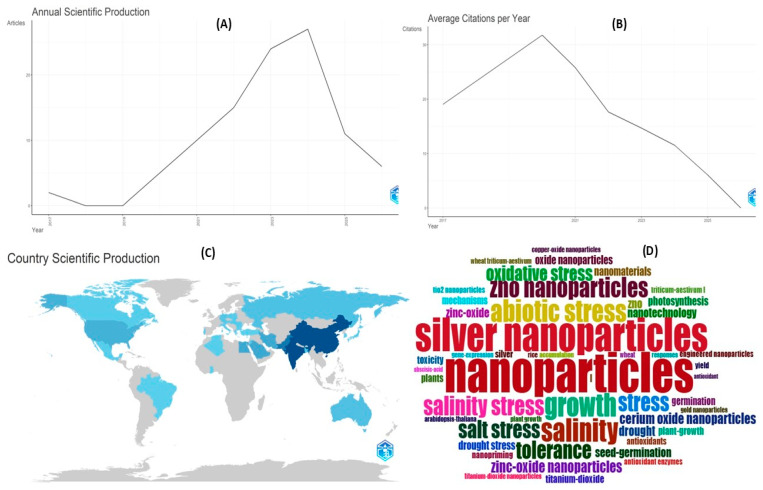
Bibliometric analysis (**A**) scientific publications, (**B**) citations, and (**C**) global research by country publication distribution and (**D**) a word cloud of keywords on nanoparticle-based approaches for enhancing salinity stress tolerance in plants (2017–2026).

The annual scientific output of studies investigating the role of nanoparticles in mitigating salinity stress is depicted in Figure 4A, which shows the publication trend of studies in this field. Publications were limited during the early years (2017–2019), followed by a steady increase from 2020. Research output peaked in 2024, reflecting growing scientific interest in using nanotechnology to improve plant performance under saline conditions. The apparent decline in 2025–2026 may reflect incomplete indexing of recent publications.

Country-wise scientific output is depicted in Figure 4C, showing the global distribution of publications. The analysis shows that Asian countries, particularly China and India, are the largest contributors to research on nanoparticle-induced salinity stress mitigation. Significant contributions are also seen from the United States, Australia, and several European and Middle Eastern countries. The geographical distribution indicates that salinity stress is being recognized as a major constraint on agricultural productivity worldwide and nanotechnology-based solutions are increasingly being adopted [40]. Darker shades indicate higher publication output. A wordcloud showing the most frequently used keywords in the analyzed literature is provided in Figure 4D. Keywords such as nanoparticles, silver nanoparticles, ZnO nanoparticles, salinity, salinity stress, salt stress, growth, tolerance, and abiotic stress dominate the research landscape. Other keywords, such as oxidative stress, seed germination, photosynthesis, nanotechnology, and antioxidant enzymes, indicate that current studies primarily focus on understanding the physiological, biochemical, and molecular mechanisms by which nanoparticles confer plant tolerance to saline environments. The size of each keyword corresponds to its frequency of occurrence in the dataset. Overall, the bibliometric analysis reveals a rapidly growing research field focused on the use of nanotechnology, especially metal and metal-oxide nanoparticles, to improve plant growth, seed germination, and stress tolerance under saline conditions. Also, NBC is an emerging field in which researchers are increasingly focusing on salinity stress management [42,43]. The growing publication output, large-scale international participation, and emphasis on physiological and molecular mechanisms demonstrate the increasing importance of NPs and other emerging materials like NBC as a new tool for sustainable agriculture in salt-affected ecosystems.

## 3. Process of Seed Germination

Seed germination can be divided into three main phases: Phase I, characterized by rapid water imbibition; Phase II, involving metabolic reactivation and cell elongation (also referred to as “chitting,” the visible onset of germination); and Phase III, marked by active cell division concurrent with radicle elongation, also termed the post-germination phase (Figure 1). Phase II represents a lag phase during which key physiological and biochemical processes are progressively reactivated, including hydrolysis of food reserves, macromolecule synthesis, respiration, reorganization of subcellular components, and cell elongation, all of which contribute to the onset of germination [44]. In monocotyledonous species, the coleorhiza is the first structure to emerge from the seed coat, whereas in dicotyledonous species, the radicle emerges first [45]. This process involves a coordinated series of physical and chemical reactions, including rupture of the testa and endosperm cap, dissolution of cellular solutes, repair of organelle membranes and DNA, and de novo synthesis of DNA, RNA, and proteins. During imbibition, gibberellins (primarily GA_3_) produced or mobilized in the embryo signal the aleurone layer to activate hydrolytic metabolism. Hydrolytic enzymes—including acid cysteine endopeptidases, serine carboxypeptidases, and neutral aminopeptidases—are subsequently activated in the endosperm, where aleurone cells degrade storage proteins into amino acids [46,47]. GA_3_ induces the expression of genes encoding α-amylase in aleurone cells through the transcription factor GAMYB (GA-induced Myb-like protein), which binds to α-amylase gene promoters and stimulates mRNA accumulation [48].

## 4. Effect of Salinity Stress on the Seed Germination Process

### 4.1. Impact of Salinity-Induced Osmotic Stress During Phase I of the Seed Germination Process

**Figure 5:**
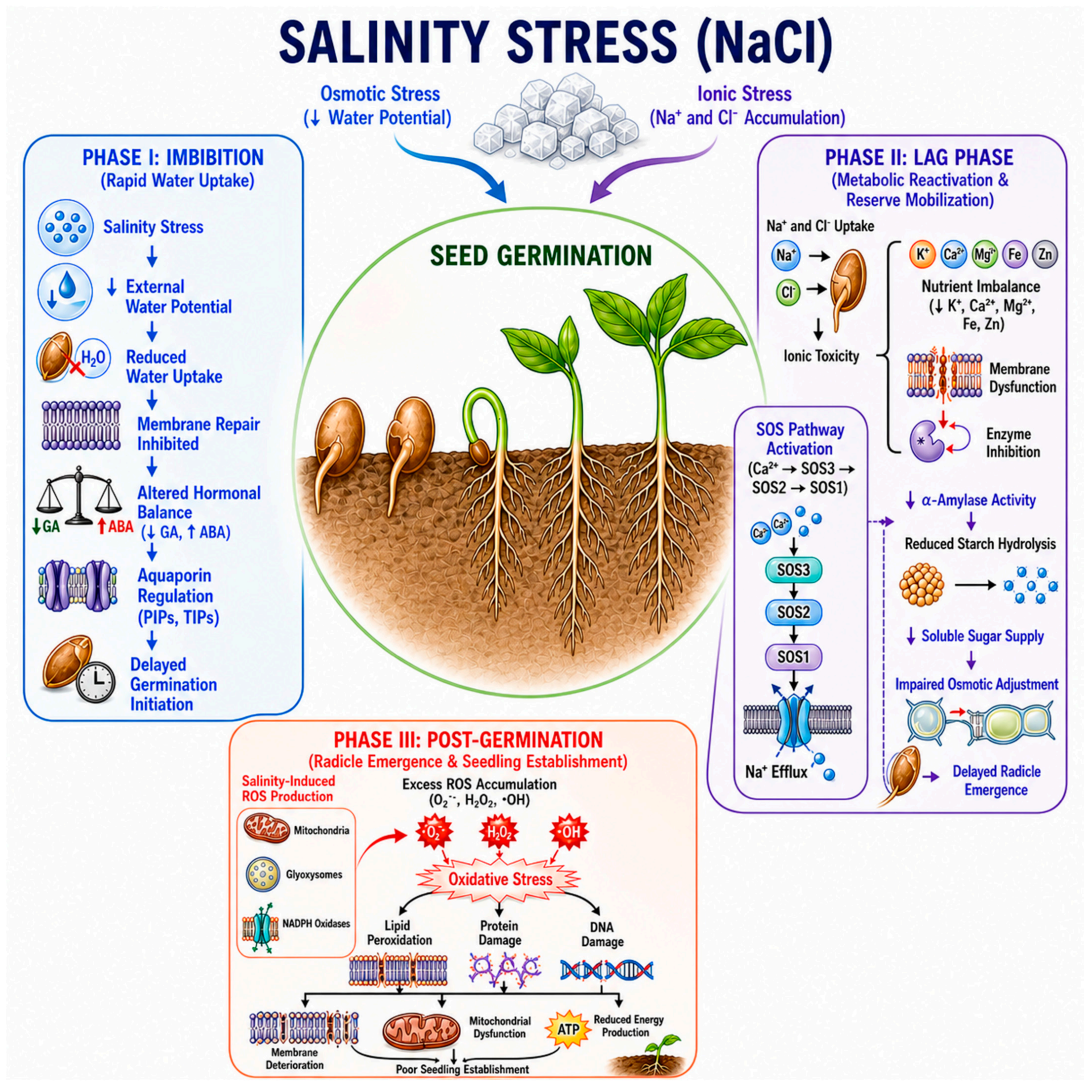
Overview of the three-phase seed germination process, illustrating key cellular and molecular events: rapid water imbibition and membrane repair in Phase I, with GA_3_-mediated initiation of the germination cascade; α-amylase activation and reserve mobilization in Phase II (Lag Phase); and cellular division, radicle emergence, and seedling establishment in Phase III (Post-Germination). GA_3_ is highlighted as a central hormonal regulator across phases, providing the mechanistic basis for understanding salinity-induced hormonal disruption. Salinity stress induces osmotic and ionic stress that negatively affects seed germination phases, along with associated biochemical and molecular alterations.

Salt stress suppresses plant water uptake and creates osmotic stress, leading to ionic imbalances through excessive Na^+^ and Cl^−^ accumulation. Successful seed germination involves water being drawn through the seed coat by diffusion and capillarity, as water moves from the region outside the seed coat (high water potential) to the region inside the seed coat (low water potential) [49]. Salt stress influences seed germination by establishing an external osmotic potential that inhibits water uptake and by causing toxic damage from Na^+^ and Cl^−^ ions on germinating seeds [50]. Water availability and imbibition into seeds are crucial for germination, the establishment of root growth, and shoot elongation [51]. Seed water absorption can be severely impaired by extremely negative water potential at the beginning of germination, further preventing seed germination [52]. The inhibitory effects of salinity on seed germination through osmotic stress have been reported in various plant species, including grass [53], *Prosopis strombulifera* [54], pepper [55] rapeseed (*Brassica napus* var. *oleifera* Del.) [56], barley and oat (*Avena sativa* L.) [57], wheat [58], purslane [59], quinoa (*Chenopodium quinoa* Willd.) [60], *Limonium* [61], Plantaginaceae [62], and *Elymus nutans* [63]. Salinity imposes osmotic stress by reducing the water potential of the germination medium relative to the interior of the seed (Figure 5) [64,65], thereby impairing water uptake into the seed. Salt stress can also negatively affect germination through ionic stress, which interferes with enzyme activity, metabolism, hormonal signaling, and energy reserve mobilization [65]. In general, the combined osmotic and ionic stress induced by salinity inhibits seed germination, delaying it and compromising crop establishment and future crop production.

Seed germination requires a variety of cellular functions, including imbibition, mRNA synthesis and translation, cell expansion, gene expression, activation of DNA repair mechanisms, and organelle reassembly, all modulated by hormonal signals and controlled by the developmental context of the seed [14,66]. Imbibition—the uptake of water into a resting, non-germinating seed—is the primary stage of germination, initiating metabolism and providing resources for protein translation, cell growth, and division processes that subsequently allow radicle emergence [66,67]. Water imbibition leads to activation of energy metabolism, modulation of oxidative status, induction of DNA repair, reactivation of the cell cycle, mobilization of energy reserves, and modification of hormonal balance [67]. It is assumed that water intake during seed imbibition occurs primarily through a narrow entrance called the micropyle [68]. Although aquaporins have been implicated in mediating water transport in seeds, the extent of membrane water movement via aquaporins relative to other mechanisms in imbibing seeds remains incompletely understood [67,69,70,71].

Aquaporins help germinating seeds adapt to unfavorable conditions such as salinity-induced osmotic stress [72,73]. Plants in saline conditions face both osmotic and ionic stress [5,74], which can prevent or delay seed germination [75,76]. Aquaporins form a large and ancient family of membrane intrinsic proteins (MIPs) that serve as channels for the passive transport of water and other solutes across membranes in plants and animals, classified into subfamilies on the basis of sequence similarity [77,78,79]. The molecular mechanism of water transport is also thought to involve aquaporins present in seed embryonic axes [80]. Tonoplast intrinsic proteins (TIPs) facilitate water influx into expanding vacuoles in developing seeds and during the conversion of vacuoles into a protein-storage type, and are thought to release water outward due to shrinkage caused by accumulated proteins [80,81,82]. Several plasma membrane intrinsic protein (PIP) isoforms have been found to affect seed germination in response to osmotic stress. For instance, suppression of OsPIP1;1 and OsPIP1;3 inhibited seed germination, while overexpression of OsPIP1;3 enhanced seed germination in a water-deficient medium [83]. The functions of OsPIPs in germination have been connected to their response to nitric oxide and reactive oxygen species signaling [84,85]. In maturing Arabidopsis thaliana embryos, transcripts of 6 out of 35 aquaporins are notably abundant, including AtPIP1;3, AtPIP1;4, AtPIP2;7/2;8, AtTIP3;1, and AtTIP3;2, while dry seeds mainly contain AtNIP1;2, AtPIP1;2, AtSIP1;1, and AtTIP3;2 [86]. AtTIP3;1 and AtTIP3;2 accumulate during seed maturation and influence germination along with AtTIP4;1 [73]. AtTIP3 isoforms modulate Arabidopsis seed germination responses to ABA, with TIP3;1 and TIP4;1 upregulated under osmotic stress [73]. During germination, the TIP3;1 transcript level decreases while PIP2;1, PIP2;7, TIP1;1, TIP1;2, and TIP4;1 increase, suggesting their involvement in post-germination cellular expansion [86]. In Zea mays, ZmPIP1s, ZmPIP2s, ZmTIP1-1, ZmTIP2s, and ZmTIP3-3 show high expression in roots during early germination compared to ACTIN 1, indicating their role in seedling growth [87]. Analysis of the Arabidopsis electronic Fluorescent Pictograph (eFP) Browser data 2.0 (available at: http://bar.utoronto.ca/) revealed that during seed imbibition and germination, aquaporins from the PIP2 subfamily are expressed from early imbibition to 48 h, particularly AtPIP2;1 [86]. These PIPs may have roles in water and hydrogen peroxide permeation, as well as monovalent cation permeation, which may depend on post-translational modifications [88,89]; however, the role of PIP2;7 in univalent cation transport remains unclear [90,91], and PIPs are also likely to be involved in signaling processes [92,93,94]. Another study hypothesizes that the phenotype of plant material with varying AtPIP2;1 expression may differ from wild-type (WT) material during germination [95]. Salinity exerts adverse effects on seed germination by decreasing gibberellic acid (GA) levels, increasing abscisic acid (ABA) levels, modifying membrane permeability, and inhibiting water uptake in germinating seeds (Figure 5) [96]. GA and ABA are two major plant hormones with established roles in the regulation of defense responses to salinity stress, and the GA/ABA balance is critically important during the initial phase of seed germination [11,97]. ABA is regarded as an abiotic stress regulator and a major player in the induction of dormancy during seed development and its maintenance during imbibition [98,99,100]. In addition, the regulation of GA metabolism is a dormancy-breaking process during after-ripening and seed germination, commonly accompanied by stimulated GA synthesis (Figure 5) [101]. Furthermore, expansin (EXP) is one of the enzymes involved in GA-induced weakening of the endospermic cap, while late embryogenesis-abundant (LEA) proteins accumulate during seed maturation and contribute to abiotic stress tolerance through ABA-dependent regulation [102].

### 4.2. Impact of Salinity-Induced Ionic Stress Predominant During Phase II of the Seed Germination Process

When seeds take up water, they simultaneously absorb toxic ions (Na^+^ and Cl^−^). This process triggers ionic stress, which tends to reach its peak during Phase II, known as the Lag Phase, as the embryo works to reactivate its metabolic functions (Figure 5) [22,103]. Ionic stress from excessive Na^+^ and Cl^−^ ions disrupts enzymes, metabolism, hormone signaling, and energy use, resulting in delayed and reduced seed germination [65]. Excessive absorption of Na^+^ and Cl^−^ leads to cellular toxicity, membrane disruption, and inhibition of seedling establishment. High Na^+^ levels reduce the availability of essential nutrients such as K^+^, Ca^2+^, and Mg^2+^ through cation competition, leading to nutrient deficiencies [104,105]. Salinity increases Na^+^ uptake relative to K^+^, disrupting ion balance and membrane function, thereby impairing nutrient assimilation [106]. Studies show that salinity decreases the concentrations of key nutrients (Fe, K, Mn, Mg, P, Zn) in roots and shoots and reduces root surface area by lowering root hair density and length, thereby further limiting nutrient uptake. Essential elements for root growth (Ca^2+^, Mg^2+^, Fe^2+^, Zn^2+^) are also diminished under salt stress, exacerbating nutrient deficiencies and growth reduction [107]. Halophyte seeds can either avoid or endure salinity stress through various strategies, such as entering dormancy or quiescence to survive unfavorable conditions, recovering after brief salinity exposure, and exhibiting seed heteromorphy [108]. Although halophytes can endure high salinity levels of up to 25 dS m^−1^ when fully grown, they may be vulnerable to elevated salinity during germination or seedling emergence [109,110,111]. In contrast, most glycophytes, including salt-sensitive species such as *Triticum*, *Zea mays*, and *Nicotiana*, lack the biochemical capability to handle salt stress effectively and cannot control xylem ion fluxes at concentrations above 4 dS m^−1^ [112,113]. Na^+^ and Cl^−^ enter roots via the symplastic pathway through a specific combination of transporters, and the surplus salt is generally sequestered into the vacuole, contributing to salinity tolerance [114,115].

The primary uptake site for Na^+^ is the root cytoplasm, from which it is subsequently transported into the vacuole by Na^+^/H^+^ antiporters [116]. Two classes of H^+^ pumps drive ion transport across the tonoplast: the vacuolar-type H^+^-ATPase (V-ATPase) and the vacuolar pyrophosphatase (V-PPase) [117,118]. Among these, V-ATPase is the most predominant H^+^ pump in plant cells and its activity is essential for plant survival under stress conditions [117,118,119]. In *Vigna unguiculata* hypocotyls exposed to salinity, V-ATPase activity increased whereas V-PPase activity was inhibited, indicating differential regulation of vacuolar ion transport systems [120]. The Salt Overly Sensitive (SOS) signaling pathway is crucial for ion homeostasis and salinity tolerance [121]. It involves three main proteins: SOS1, a Na^+^/H^+^ antiporter regulating Na^+^ efflux; SOS2, a serine/threonine kinase activated by Ca^2+^ signals; and SOS3, a myristoylated Ca^2+^-binding protein [122,123]. Increased intracellular Na^+^ raises Ca^2+^ levels, which bind to SOS3. SOS3 then activates SOS2, forming a complex that phosphorylates SOS1 on the plasma membrane, promoting Na^+^ efflux and reducing cellular Na^+^ concentration [124].

α-Amylase is a key hydrolytic enzyme involved in reserve mobilization during seed germination, secreted into the endosperm where it breaks down reserve starch into digestible sugars, providing metabolically available energy to the embryo, plumule, and radicle [125,126]. While the final germination percentage of most crops may not be affected by salinity stress, a significant delay in germination time commonly occurs. Delayed water uptake and reduced α-amylase activity contribute to the prolonged germination time observed under salinity stress (Figure 5) [125,126]. The reduction in α-amylase activity with increasing NaCl concentration has been observed across multiple studies and is more severe in salt-sensitive genotypes than in salt-tolerant genotypes. This decrease results in reduced sugar transport to the growing embryo, thereby raising the osmotic potential of growing cells and restricting water uptake, causing delayed seed germination [127,128,129,130,131,132,133]. Since the primary response of seeds in saline soil is osmotic stress, α-amylase-mediated degradation of reserve starches into soluble sugars is essential for osmotic adjustment. Elevated soluble sugar concentrations decrease the loss of turgidity in germinating cells and may allow seeds to absorb water from saline soils while providing the energy needed for germination [127,134,135]. Mobilization of reserve metabolites, including starch and proteins, has been documented to occur on a large scale during late Phase II [136,137,138,139]. Additionally, some defense proteins, such as the *salT* gene product, increase during Phase II to protect germinating seeds from salinity stress [137]. In late Phase II and Phase III, genotypic differences become significant, with seed fresh weight increasing under saline treatments [140]. Overall, salt tolerance during germination in wheat appears to be mediated by the regulation of water uptake and α-amylase activity, which together drive the hydrolysis, degradation, and mobilization of seed storage reserves required for germination and subsequent radicle and plumule growth [140].

### 4.3. Impact of Salinity-Induced Oxidative Stress Accumulating Throughout Phase III (Post-Germination)

ROS play dual roles during seed germination, functioning in signaling at controlled levels but causing oxidative damage when excessively accumulated under salinity stress [141,142]. Understanding the baseline ROS dynamics during normal seed aging is essential for contextualizing the amplified oxidative damage imposed by salinity stress, as both processes converge on the same mitochondrial ROS-generating machinery and antioxidant defense systems. Within hydrated seeds, the mitochondria, glyoxysomes, and plasma membrane NADPH oxidases are the primary sites for ROS generation (Figure 5) [141]. ROS may also accumulate in dry seeds due to nonenzymatic reactions [143]. They are produced by incomplete reduction of oxygen, giving rise to superoxide anions (O_2_^−^·), hydrogen peroxide (H_2_O_2_), and hydroxyl radicals (·OH). It has been speculated that an increase in intracellular ROS levels could be responsible for processes that lead to the decay of seed viability [144,145,146]. Following the *‘free radical theory of aging’* [147], the decline in seed viability during senescence is brought about by enhanced ROS (O_2_^−^·, H_2_O_2_, and ·OH) production [146], a deficit in antioxidant potential within cells [148], and progressive oxidative damage to seed cell molecules that accumulate over time. These assumptions have been confirmed in many studies of aged seeds [146,149,150,151,152,153]. ROS react with lipids to form oxides and inactivate enzymes, altering the structure–activity relationships of proteins and carbohydrates and modifying or breaking DNA chains [12]. One hypothesis is that lipid peroxidation is the principal mechanism of seed aging, as changes in membrane permeability due to lipid peroxidation cause a decline in seed viability [146]. The greatest oxidative damage during seed aging occurs in mitochondria, since they are the major source of ROS generation and are therefore subjected to rapid and intense oxidative damage compared with other cellular structures [153,154,155,156,157]. During natural aging of beech (*Fagus sylvatica* L.) seeds, a several-fold increase in H_2_O_2_ content was observed in mitochondria from embryonic axes and cotyledons [146]. Mitochondrial oxidative phosphorylation is one of the major cellular ROS producers, and therefore the free radical theory of aging can be regarded as a mitochondrial theory of aging in plant seeds [157]. Furthermore, ROS accumulate in the mitochondria, resulting in dysfunction of mitochondrial membranes and oxidative damage to mitochondrial proteins and DNA [158], ultimately leading to inhibition of phosphorylation [159]. During seed aging, excessive ROS generation in mitochondria progressively overwhelms the antioxidant system, with decreased activity in the ascorbate–glutathione cycle and in enzymatic antioxidants reported by several authors [154,155,156]. The interactive effects of salt stress and water deficit mediate a wide range of negative impacts on plant metabolic processes, generating ROS that affect the functioning of many physiological systems in plants. Salt-induced oxidative stress has been documented in numerous crops, including wheat [160], rice [161,162], maize [163], tomato [164], mung bean [165], mulberry [166], pumpkin [167], soybean [168], *Brassica oleracea* var. *capitata* L. [169], *Chamerion angustifolium* (L.) [170], fenugreek [171], and melon [172]. Although seeds possess enzymatic (e.g., superoxide dismutase, catalase, and peroxidases) and non-enzymatic antioxidant systems to combat ROS, these defenses may be overwhelmed under continuous or high salinity [173,174,175]. For instance, in maize seedlings, oxidative stress and certain antioxidant-mediated defensive responses are induced by salinity stress [174]. Strategies to improve antioxidant activity, such as nanopriming treatment, have been reported to be effective in alleviating the detrimental effects of salinity on seed germination by eliminating ROS and regulating the native antioxidant system [176,177,178,179,180,181,182,183,184,185]. Under salinity stress, this mitochondrial ROS machinery is further dysregulated, compounding the oxidative burden beyond what seeds can manage through endogenous antioxidant systems. Collectively, excessive ROS accumulation during post-germination growth disrupts membrane integrity, energy metabolism, and antioxidant homeostasis, thereby compromising seedling establishment under salinity stress. The key morpho-physiological, biochemical, and molecular effects of salinity stress on seed germination discussed across Section 4.1, Section 4.2 and Section 4.3 are summarized in Table 1.

Hormesis in plants exhibits a biphasic dose response, where low stressor or toxin levels are beneficial, while high levels cause inhibition or toxicity [216,217,218]. Hormesis-induced priming is an environmental signal that primes plants, enabling a rapid and robust response to a stressor [216,219,220]. This approach takes advantage of the plant’s natural defense mechanisms against environmental challenges [221]. Recently, nanopriming has gained attention for its potential to improve seed germination, plant adaptation, and productivity [222]. Nanopriming with biogenic ZnO and Cu nanoparticles influences black gram (*Vigna mungo*) germination and plant vigor by showing a hormetic pattern. At low concentrations, redox-active metal ions serve as signaling triggers, enhancing growth, while at higher levels, they disrupt oxidative balance [223]. Biosynthesized TiO_2_NPs, coated with phytochemicals from *Zygophyllum simplex* extract, enhance faba bean (*Vicia faba* L.) seed germination and related metrics. The findings indicate that TiO_2_NPs exhibit hormetic behavior: at low concentrations, they stimulate metabolic processes associated with stress response and energy utilization, while higher doses disrupt seed homeostasis [224].

## 5. Synthesis, Uptake, and Transport of NPs and NBC

**Figure 6:**
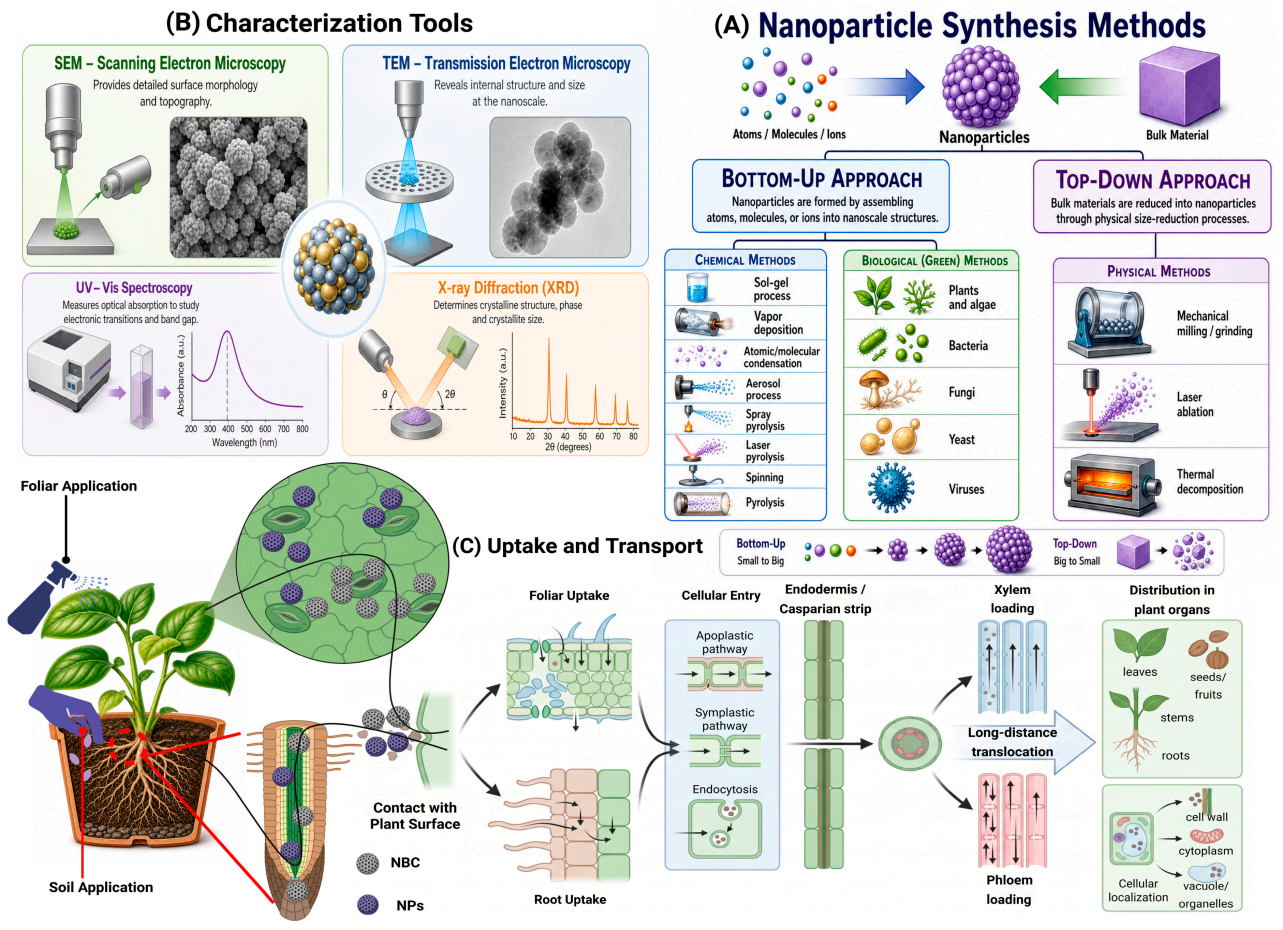
(**A**) Classification of nanoparticle (NP) synthesis methods, including bottom-up (chemical and biological) and top-down (physical) methods. (**B**) Common characterization techniques used for NP analysis, including microscopy (SEM, TEM) and spectroscopic methods (UV–Vis spectroscopy and XRD) to determine particle size, morphology, and structural properties. (**C**) Schematic overview of the uptake, transport, and fate of NPs and NBCs in plants, showing root and foliar uptake pathways, translocation through the xylem and phloem, and distribution in different plant tissues and cellular compartments.

NP synthesis methods are generally divided into two main approaches: bottom-up and top-down (Figure 6A) [225]. The bottom-up method involves creating nanoparticles from atoms, molecules, or small building blocks through a controlled nucleation and growth process. This method is widely used because it allows for precise control over particle size, shape, and composition. Bottom-up synthesis can be further divided into chemical and biological methods [226]. Chemical synthesis methods are among the most commonly used technologies for NP production [227]. These methods include spinning, pyrolysis, sol–gel synthesis, vapor deposition, atomic or molecular condensation, aerosol processes, spray pyrolysis, and laser pyrolysis [228]. Chemical methods offer high production efficiency and enable the synthesis of NPs with specific physicochemical properties suitable for various industrial, environmental, and biomedical applications. Biological synthesis methods, also known as green or biogenic synthesis, use living organisms or their metabolites to produce NPs [229,230]. Biological agents such as plants, algae, bacteria, fungi, yeast, and viruses can act as reducing and stabilizing agents during NP formation [230]. These methods are environmentally friendly, inexpensive, and reduce the use of hazardous chemicals, making them more attractive for sustainable nanotechnology applications [231]. In contrast, top-down approaches break down bulk materials into nanoscale particles through physical processes [225,228]. This approach starts with larger materials and reduces their size to the nanoscale. Common physical methods used in top-down synthesis include mechanical milling, laser ablation, and thermal decomposition [225]. These techniques are effective for producing NPs in large quantities; however, they can require significant energy input and sometimes result in a wider distribution of particle sizes than bottom-up methods [232]. Overall, both bottom-up and top-down approaches play a vital role in NP synthesis. Choosing the right synthesis method depends on the desired NP properties, production scale, cost, and intended application. While bottom-up methods offer greater control over NP morphology and composition, top-down methods offer practical advantages for large-scale production. Characterization tools, including microscopy methods such as SEM and TEM and spectroscopic methods such as UV-Vis spectroscopy and X-ray diffraction, are used to analyze the size, texture, and structural characteristics of NPs (Figure 6B) [232].

The transport of NPs within plants is a complex process influenced by many factors. NPs can enter the plant system through absorption sites such as leaves and roots (Figure 6C) [233]. Once NPs are absorbed, they can enter cells via osmosis for intracellular transport and travel throughout the vascular system to interact with plant tissues [234]. (A) Root uptake via soil application: Roots are a major route for NP uptake in plants. NPs can be absorbed through several pathways, including diffusion in the soil solution, adsorption from water released by plant roots, and osmosis through cell walls. The review cites several papers showing that NP size, surface charge, and surface modification of NPs affect uptake efficiency and the kinetics of root uptake into the plant system [235,236]. (B) Leaf absorption via foliar application: NPs can penetrate the plant through stomata, crevices within the cuticle or adsorption on the leaf surface, and when NPs are applied with the foliar method, they facilitate penetration into the plant [237,238,239].

**Figure 7:**
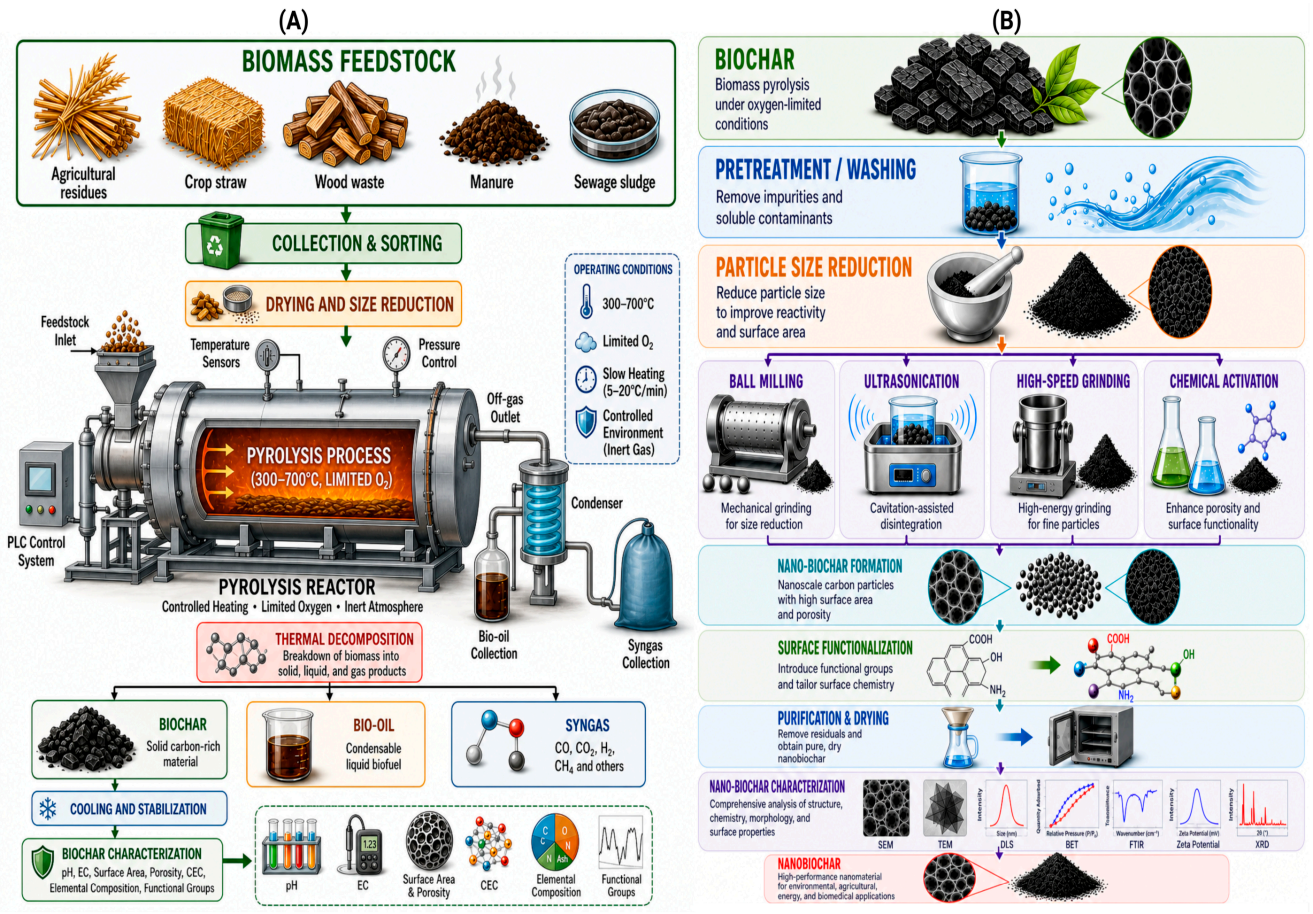
Schematic representation of the production methods for (**A**) biochar and (**B**) nanobiochar. (**A**) Biochar is produced by pyrolysis of biomass feedstocks, including agricultural residues, crop straw, wood waste, and manure, under oxygen-limited conditions, followed by cooling, stabilization, and characterization. (**B**) Nanobiochar is produced from bulk biochar by particle size reduction techniques such as ball milling, ultrasonication, high-speed grinding, or chemical activation, followed by surface functionalization, purification, and physicochemical characterization. The resulting nanobiochar has a larger surface area, better porosity, and higher surface reactivity, making it suitable for use in agriculture for salinity stress management.

Biochar can be produced by several methods, including hydrothermal, high-temperature, and flash pyrolysis and the chemical and physical properties of biochar’s surface are directly affected by the type of feedstock, the method of carbonization, and the temperature (Figure 7A) [240]. Pyrolysis temperature is an important factor affecting the surface area and pore structure of biochar [241]. Generally, when biochar is heated, its pore-expanded surface area increases relative to its original surface area [242]. Extremely high temperatures (such as those that cause loss of functional groups or increased ash formation) can alter biochar properties in several ways [243]. Pyrolysis biochar produced at higher temperatures has higher porosity than thermal carbonization biochar [244]. Nanobiochar is a new form of biochar that incorporates nanomaterials with particle sizes in the micron-meter range, giving it unique nanoscale dimensions [245]. Nano-synthesis also increases the surface area and reactivity of the particles, improving nutrient absorption mechanisms [246].

A common process for producing NBC from biomass is shown in Figure 7B, which takes feedstocks and converts them into biochar at harvest scale. To achieve this, harvested bulk biochar is milled to nanoscale sizes by means of various physical methods, e.g., pyrolysis, ball milling, centrifugation, or dispersion by ultrasonication [247]. Therefore, it is cost-effective due to its texture and serves as a large-scale synthesis step for NBC production. Ball milling is one of the most common green and efficient methods, in which biochar is broken into NPs by metal or zirconia balls propelled at high speeds and subjected to thorough fractural collisions [245]. Parameters such as ball diameter, weight, grinding time, and rotation speed can be used to fine-tune particle size with high precision [248]. Two methods have been used: dry and wet ball milling. Wet ball mills produce NBC with a larger specific surface area than dry ball mills, with smaller particle sizes, less separation of powder within inclusions, and smaller pore sizes [249]. Yuan et al. [250] showed that ball milling can nearly double the surface area of NBC, while the average pore size of NBC decreased from 23.2 nm to 6.4 and 6.25 nm under dry and wet conditions for 2 h, respectively. Ultrasonic treatment is another effective method [250]. Dong et al. experimented with ultrasonicated tobacco-derived biochar, producing NBC with a particle size of 42 ± 6 nm and a surface area of 21.1 m^2^/g [251]. Centrifugation is also an effective method for classifying smaller particles by setting the centrifugal force. This separation of biochar particles from micron to nanoscale occurs due to the high gravitational force of multi-stage centrifugation [249,252].

For clarity in the sections that follow, the nanomaterials reviewed here are grouped not only by synthesis route, as set out above, but also by material class and by mode of application—a distinction that is mechanistically consequential and that the primary literature tends to blur. Five classes of nanoparticle are applied predominantly as seed-priming agents: metal-oxide NPs (ZnO, TiO_2_, Fe_2_O_3_, MnO/Mn_2_O_3_); metalloid and rare-earth NPs (SeNPs, CeO_2_/nanoceria, including polyacrylic acid-coated nanoceria, PNC); metallic NPs (Ag NPs); silica NPs (SiO_2_/Si NPs); and carbon-based NPs (CNPs). Two carbon-based classes are applied predominantly as soil amendments or substrate incorporations: nanobiochar (NBC) proper, whether pristine, ball-milled or chemically activated; and metal-modified (nano-)biochars, in which biochar is combined with metal-oxide NPs (ZnOBC-20, ZnBC@2, FeBC-10/FeBC@1, SBC).

## 6. Effect of NPs and NBC on Seed Germination Indices Under Salinity Stress

The germination percentage (GP%) is the proportion of seeds that successfully germinate relative to the total number of seeds sown [15,253]. Germination rate (GR) indicates how quickly germination occurs and may vary depending on several factors, such as seed size, natural environmental conditions, and seed pre-treatment [15]. The Germination Index (GI) is a measure of the rate and completeness of seed germination, applying a weighted sum of daily germination counts with greater weight given to earlier germinators; a higher GI indicates rapid and complete germination [16,17]. Mean Daily Germination (MDG) is defined as the average number of seeds germinating per day during a germination test, with higher values indicating faster germination and potentially higher seed quality [18,19]. Mean Germination Time (MGT) reflects the average time required for seed germination within a population; seeds with a lower MGT germinate faster, while a higher MGT indicates delayed germination [16,20]. Germination Energy (GE) is expressed as the percentage of seeds that germinate within an early time point—typically the fourth day—under optimal conditions, serving as an indicator of seed vigor and uniformity of emergence [21]. All of these indices are negatively affected by salinity stress [18,21,22,23,24,25].

### 6.1. Effect on Seed Germination Percentage (GP%) and Germination Rate (GR)

Under non-saline conditions, the GP of all sorghum genotypes was 100%. Despite a 100 mM NaCl increase in salinity, genotypes PEGAH, GS4, JUMBO, KGS23, and SPEED FEED showed no change in GP, whereas reductions of between 5% and 9% were recorded in genotypes MGS5, KIMIA, KGS29, SEPIDEH, and PAYAM. GP was significantly decreased in all sorghum genotypes under 150 and 200 mM NaCl. Under 200 mM NaCl, the highest reductions in GP compared to the non-saline condition belonged to genotype PAYAM (49.9%), and the lowest reductions were recorded for genotypes PEGAH (33%), GS4 (34.4%), and JUMBO (33.4%), confirming their classification as the most salt-tolerant genotypes [24].

In wheat, 50 mM and 100 mM NaCl solutions reduced GP% by 77.23% and 79.54%, respectively, with further reductions of 87.01% and 89.33% recorded at higher salinity levels. In contrast, application of 50 µg/mL TiO_2_ NPs recovered GP% by 72.26% in Pirsabak-05 and 68.11% in NARC-09 at 50 mM NaCl, and by 65.04% and 59.21%, respectively, at 100 mM NaCl, compared to the salt-stressed unprimed control [254]. The GP% of tomato seeds was determined primarily by cultivar type but was markedly influenced by ZnO NPs’ concentration and size. At 250 mg/L, robust variation was noted in the Granit cultivar, and statistical analysis revealed that ZnO NPs did not impede seedling germination. Malinowy Bosman recorded the highest GP (>90%) at all concentrations when treated with <50 nm ZnO NPs, while Granit recorded the lowest GP (74.6%) at 250 mg/L with <100 nm ZnO NPs. It should be noted that this study was conducted under non-saline conditions; the reported results therefore reflect ZnO NPs’ phytotoxicity thresholds rather than salinity mitigation effects [255]. In a nanopriming experiment evaluating the effects of SeNPs and ZnO NPs on early seedling responses to salt stress, germination time and final germination percentage (FG%) did not differ significantly between primed seeds and the control but differed significantly under NaCl stress. By contrast, the GR of salinity-stressed unprimed seeds decreased by 21.82% (Yangyou 9) and 40.66% (Zhongshuang 11) compared with the control. Yangyou 9 showed a significantly higher GR of 42.16% and 3.07% under SeNPs, and 49.20% and 8.18% under ZnO NPs, while Zhongshuang 11 showed even larger GR increases of 167.2% and 23.60% under SeNPs, and 171.4% and 25.53% under ZnO NPs, seven days after sowing under salinity stress compared with NaCl and hydropriming (HP) treatments, respectively [256]. In another experiment, polyacrylic acid-coated nanoceria (PNC) combined with TES (trisodium EDTA solution, used as vehicle control) priming recorded a final germination rate of 84%, significantly higher than TES priming alone (76%) under 200 mM salinity stress over seven days [257]. Under salt stress conditions, salicylic acid (SA) priming increased rapeseed seed germination by 8% (82 ± 1.5 vs. 72 ± 1.1) compared with the control, while PAC (salicylic acid biosynthesis inhibitor) treatment inhibited germination even under normal conditions, yielding only 76% germination compared to 96% (SA+PAC) and 86% (PNC+PAC). Under saline conditions, SA+PAC and PNC+PAC enhanced rapeseed germination over PAC treatment alone by 226% and 198%, respectively [258]. Application of NBC at four levels (0%, 1%, 3%, and 5%) under salt stress (80 mM NaCl) and non-stress conditions (0 mM) showed that salt stress decreased radicle length, plumule length, and seedling weight. At all concentrations tested, 5% NBC significantly improved growth and germination indices of wheat seeds [35].

### 6.2. Effect on Germination Index (GI)

Salinity stress significantly reduced the GI of sorghum (*Sorghum bicolor* (L.) Moench). The highest reductions under salinity were recorded in genotype PAYAM (from 21.5% at 100 mM to 49.9% at 200 mM NaCl) and genotype SEPIDEH (from 19.7% to 42.9%, respectively), while the lowest reductions were observed in salt-tolerant genotypes PEGAH (from 10.9% to 33%), GS4 (from 11.9% to 34.4%), and JUMBO (from 14.7% to 33.4%) [24]. Application of NPs enhanced the GI of seeds under salinity stress. In wheat, exposure to 50 mM and 100 mM NaCl reduced GI by 70.76% and 74.31% and by 81.14% and 85.24%, respectively, in two varieties (Pirsabak-05 and NARC-09), while 150 mM NaCl resulted in total absence of germination in both varieties. Application of 50 µg/mL TiO_2_ NPs recovered GI by 67.28% and 61.35% at 50 mM NaCl, by 61.14% and 49.33% at 100 mM NaCl, and by 30% and 27% at 150 mM NaCl, in Pirsabak-05 and NARC-09, respectively [254]. Additionally, application of 5% NBC has been shown to positively influence GI under salinity stress [35].

### 6.3. Mean Daily Germination (MDG)

Salinity decreased seed germination, presumably due to both osmotic and ionic toxicity, and thus reduced MDG (Table 1). Salt-susceptible genotypes are generally more affected than salt-tolerant genotypes across different agronomic traits under salt stress [259]. Among Iranian ecotypes, Yazd, Arak, Bandarabas, and Kermanshah exhibited the greatest MDG reductions at high salinity concentrations (8 and 12 dS m^−1^) compared to the control and were therefore classified as salinity-sensitive. In contrast, Lorestan, Damavand-Tehran, Shiraz, Tehran, Tehran-Karaj, Khash-Sistan, Rodan-Hormozgan, Khoramabad, Kashan, and Isfahan ecotypes showed the least MDG reduction at the same salinity levels and were classified as salinity-tolerant [260]. In eight durum wheat cultivars (Karim, Khiar, Inrat100, Maali, Monastir, Portodur, Razeg, and Salim) treated with NaCl at 0, 2, 4, 6, and 10 g L^−1^, MDG was markedly influenced regardless of NaCl concentration; Karim and Salim remained the least affected, followed by Inrat, Khiar, Monastir, and Portodur, whereas Razeg and Maali were the most affected [18]. An experiment on tomato plants showed that under salinity stress (50 and 100 mM NaCl), MDG was reduced by up to 12.6% and 14.1%, respectively, but application of ZnO NPs at 750 ppm for 6 h improved MDG under both salinity levels [261].

### 6.4. Mean Germination Time (MGT)

Higher MGT values are associated with delayed germination due to seed dormancy, stress conditions such as salinity, or poor seed quality [262]. The MGT parameter increased by 2 to 4% in all genotypes under moderate salinity stress (MSS, 5 g L^−1^ NaCl) but was significantly higher under severe salinity stress (SSS, 10 g L^−1^ NaCl), with increases of +31% in Karim, +22% in Razeg, and +17% in Maali. When primed with ZnSO_4_ or KNO_3_, MGT was partially alleviated only in Karim (+24% and +18%, vs. +31% without priming under SSS). By contrast, priming did not mitigate the SSS-induced germination delay in Razeg or Maali; MGT continued to increase (+53% and +44% in Razeg; +25% and +23% in Maali), indicating that the efficacy of priming is strongly cultivar-dependent [263]. These results further demonstrate that MGT responses to priming cannot be generalized across genotypes. High salinity stress (6–10 g L^−1^ NaCl) increased MGT in wheat, resulting in concurrent reductions in GR, final germination percentage (FG%), GI, and MDG [18]. In wheat, nano-Se reduced MGT at all tested concentrations (50, 75, and 100 mg L^−1^) under the highest NaCl concentration (150 mM). The shortest MGT (4.5 days) was recorded in seeds treated with 75 mg nano-Se L^−1^, followed by 100 mg nano-Se L^−1^ (4.7 days), while control seeds showed the longest MGT (5.4 days) at 150 mM NaCl [264]. Application of Si NPs (50 and 100 nm) at 100 mM NaCl reduced MGT and improved seed germination in wheat by breaking dormancy, thereby enhancing subsequent growth [265]. Overall, MGT represents a sensitive and informative index for assessing NP- and NBC-mediated improvements in germination speed and uniformity under salinity stress.

### 6.5. Germination Energy (GE)

In purslane (*Portulaca oleracea* L.), GE decreased significantly under salt treatment at 50, 100, 200, and 400 mM NaCl, dropping sharply from 91.55% in the control to 4.79% in salt-stressed seeds, demonstrating the severe inhibitory effect of salinity on early germination vigor [59]. Nanopriming can partially counteract this suppression; seed priming with ZnO NPs effectively improved GE of maize (*Zea mays* L.) seeds under saline conditions (50 and 100 mM NaCl) [266].

**Figure 8:**
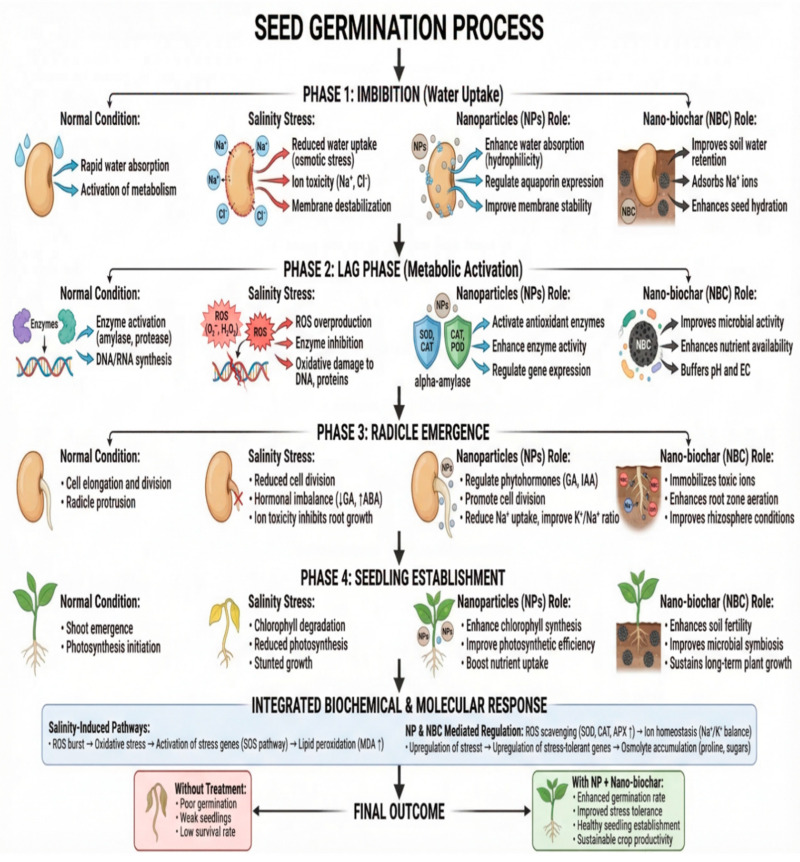
Overview of nanoparticle- and nanobiochar-mediated mitigation of salinity stress across the three phases of seed germination, shown in direct correspondence with the salinity-induced constraints depicted in Figure 1. NPs act predominantly at the seed level, enhancing water uptake through aquaporin upregulation (PIP and TIP isoforms), restoring ROS homeostasis through induction of SOD, CAT, POD and APX, promoting reserve mobilization via α-amylase (AMY1, AMY2), and shifting the ABA/GA balance toward germination. NBC acts predominantly through the soil, improving water retention, adsorbing Na^+^ and maintaining the K^+^/Na^+^ ratio, and enhancing nutrient bioavailability, with downstream induction of antioxidant and stress-responsive genes. Morpho-physiological outcomes are shown in the outer ring, biochemical responses in the middle, and molecular targets at the center.

Overall, the effects of NPs and NBC on germination indices discussed across Section 6.1, Section 6.2, Section 6.3, Section 6.4 and Section 6.5 are underpinned by coordinated morpho-physiological, biochemical, and molecular mechanisms, as summarized in Figure 8.

## 7. Mechanistic Effects of NPs and NBC on Seedling Morpho-Physiology, Biochemical, and Molecular Traits in Response to Salinity Stress

### 7.1. Morpho-Physiology Responses of Seedlings to NPs and NBC Under Salinity Stress

Salinity stress severely impairs seedling establishment by disrupting water relations, ion homeostasis, cellular integrity, and photosynthetic performance (Figure 4) [267]. Reduced growth constitutes a primary symptom of salt stress, underscoring the importance of selecting tolerant cultivars in saline environments [268,269]. Higher osmotic and ionic imbalances under salt stress reduce shoot length and leaf area due to decreased cell division and elongation [270], with a dose-dependent decline in growth-related traits observed as salinity intensity increases [271]. Salt stress also inhibits root growth by disrupting osmotic and ionic balance, generating oxidants and free radicals that alter membrane potential, integrity, and permeability; it also hampers photosynthesis and protein synthesis in *Brassica* seedlings [112,269].

Nanopriming with various NPs has been shown to mitigate these effects and improve seedling morpho-physiological performance under salinity. In rapeseed (*Brassica napus* L.), ZnO NP priming under 150 mM NaCl increased shoot length by 9.60% and 25.63% and root length by 41.64% and 48.17% in Yang You 9 and Zhong Shuang 11, respectively, compared to hydropriming, with considerable elevation in biomass [272]. Similarly, ZnO NPs at 200 mg L^−1^ improved seedling growth and biomass in rice under salinity levels of 0, 1.5, and 3% [273]. Residual effects of nano-modified biochar (FeBC-10 and ZnOBC-20) significantly promoted rice plant growth under saline conditions by increasing shoot and root biomass [274], with similar findings reported in soybean [275], rice [276], and wheat [277]. Under salinity stress, growth reductions in rice are linked to decreased water availability and Na^+^ toxicity; the rice cultivar Jing Liang You-534 exhibited greater reductions in shoot height, fresh weight, and dry weight relative to Xiang Liang You-900, indicating higher susceptibility to salt stress likely due to disrupted cellular homeostasis [278].

High salt stress damages cellular membranes and impairs chloroplasts, thereby decreasing chlorophyll content, limiting CO_2_ availability, degrading photosynthetic pigments, and impairing the activity of photosystem II and electron transfer rates [276,279], with a consequent reduction in the plant’s overall photosynthetic capacity [278]. Salt stress also reduces gas exchange parameters—net photosynthesis (Pn), stomatal conductance (Gs), transpiration rate (Tr), and intercellular CO_2_ concentration (Ci)—directly altering chloroplast structure and leading to stomatal closure and photo-inhibition [280]. Furthermore, plants under sodium stress show significant changes in chlorophyll fluorescence properties, including the maximum quantum efficiency of photosystem II (Fv/Fm), electron transport rate (ETR), non-photochemical quenching (NPQ), and photochemical quenching coefficient (qP) [281]. Reduced K^+^ accumulation and a decreased K^+^/Na^+^ ratio directly affect stomatal parameters, lowering ETR and disrupting PS-II, with consequent declines in chlorophyll fluorescence and photosynthetic rates under increasing salinity [282,283].

Under salinity stress, NPs exert a protective role in seedlings primarily by restoring chlorophyll levels and preventing oxidative damage to the photosynthetic machinery [284,285]. Previous studies have demonstrated that NPs can restore photosynthetic function in *Helianthus annuus* [286], *Triticum aestivum* [284], *Oryza sativa* [285], *Zea mays* [287], and *Punica granatum* [288]. The effects of biochar application include enhanced chlorophyll content, improved water use efficiency, and better stomatal regulation under water deficit and salinity stress, all of which support photosynthesis [264,289]. Biochar facilitates increased nutrient uptake and enhanced plant growth by improving soil water retention and nutrient availability, thereby alleviating stomatal injury and boosting electron transport in photosystem II [289,290]. Residual effects of ZnOBC-20 in the soil promote ongoing adaptation of plants to saline environments even after application ceases, as the gradual release of nutrients enhances their bioavailability for plant uptake [43]. This adaptation also helps protect cell membranes and supports improved plant–water relations under saline stress through osmotic regulation [291,292], with previous findings demonstrating a significant increase in relative water content (RWC) following ZnOBC-20 residual application [293]. Furthermore, ZnOBC-20 residues markedly increase osmolyte levels alongside enhanced K^+^ uptake, contributing to the maintenance of the K^+^/Na^+^ ratio and the regulation of stomatal activity, enzyme function, PS-II stability, and ion homeostasis [293]. Collectively, these mechanisms form a protective barrier against salt stress, enhancing CO_2_ uptake and photosynthetic efficiency, with subsequent improvements in rice growth attributes including plant height under salt stress [278].

### 7.2. Biochemical Responses of Seedlings to NPs and NBC Under Salinity Stress

Salinity stress disrupts photosynthetic electron transport, leading to excessive ROS generation and oxidative membrane damage, commonly reflected by elevated malondialdehyde (MDA) accumulation (Figure 4). Salt-stress-induced overproduction of ROS in chloroplasts and mitochondria results from disrupted electron transport chains and causes subsequent oxidative damage to cellular components such as lipids, proteins, and nucleic acids, thereby hindering plant growth [282,294]. TiO_2_ nanopriming at 60 ppm improved maize seedling germination and growth under salinity stress, with significant increases in germination percentage, germination energy, seedling vigor index (SVI), root and shoot lengths, fresh and dry weights, potassium ion concentration, relative water content (RWC), proline, and total phenolics, as well as SOD, CAT, L-reductase, and nitrate activity. Conversely, mean emergence time (MET, a field-based analog of MGT measured at seedling emergence), sodium ion concentrations, membrane electrolyte leakage (MEL), and MDA were significantly lower in TiO_2_-primed plants compared to controls [295]. Seed priming with carbon nanoparticles (CNPs) at 80 mM under mild salinity conditions (25 mM NaCl) enhanced radish sprouting and antioxidant activity, activating metabolites such as polyphenols, flavonoids, polyamines, anthocyanins, and proline [296]. The application of ZnO NPs increased the levels of CAT, peroxidase (POX), and polyphenol oxidase (PPO) antioxidant enzymes, enhancing the salinity tolerance of peas (*Pisum sativum* L.) [297]. Combining ZnO NPs with BC enhanced antioxidant defenses and effectively reduced ROS-induced oxidative damage under stress [298]. In rice, ZnOBC-20 treatment significantly increased the activities of SOD (10% and 10.9%), POD (11.6% and 19.7%), CAT (17.1% and 9.7%), and APX (12.9% and 6.9%) across both genotypes compared to FeBC-10 [293]. Similarly, the nano-modified biochar formulations FeBC@1 and ZnBC@2 led to significantly greater increases in SOD, POD, CAT, and APX compared to conventional BC under salt stress [293]. The SiO_2_-biochar formulation (SBC) decreased H_2_O_2_ and MDA levels in rice plants, indicating reduced oxidative stress and improved cellular integrity, likely due to increased activity of SOD, CAT, and POD [299].

ZnBC@2 significantly enhanced antioxidant enzyme activity, mitigated oxidative injury, and increased the salt stress tolerance of rice plants [293]. Salt stress often increases total soluble protein accumulation as part of osmotic adjustment and stress adaptation, and nano-modified biochar further enhances protein production in salt-stressed plants, possibly through increased enzymatic activity [300]. Proline, an osmoprotectant amino acid that stabilizes proteins and membranes under dehydration stress [301], accumulated to higher levels following ZnBC@2 application, contributing to increased stress tolerance. This effect may be attributed to the upregulation of pyrroline-5-carboxylate synthase (P5CS), a key enzyme in proline biosynthesis [302]. Additionally, ZnBC@2 stimulated enzyme activity in glycolytic and pentose phosphate metabolic pathways, leading to the accumulation of total soluble sugars as osmolytes for osmotic adjustment under stress conditions [283]. Some organic compounds in biochar may also be absorbed by rice plants and interact with ABA-relevant proteins to modulate the ABA signaling pathway, further increasing stress tolerance [303]. Collectively, these metabolic modulations mediated by NPs and NBC—including enhanced synthesis of total soluble proteins, proline, and soluble sugars, alongside increased antioxidant enzyme activity—stabilize ROS balance, reduce oxidative damage, and improve plant salt tolerance and productivity.

### 7.3. Molecular Responses of Seedlings to NPs and NBC Under Salinity Stress

The application of NPs, BC, and NBC enhances seed germination through several mechanisms: upregulating aquaporin and hydrolytic enzyme genes, preserving ROS homeostasis, regulating antioxidant enzyme genes, and modulating GA and ABA biosynthesis gene expression while maintaining ion homeostasis (Figure 4). Aquaporins are the primary targets for regulating water exchange and are influenced by membrane modifications and osmotic imbalance [304], playing essential roles in salt stress responses across multiple crops [304,305]. PIPs regulate the majority of water flow in and out of cells and are strongly linked to overall plant hydraulic conductance, while TIPs facilitate cytoplasmic–vacuolar exchange, adjusting cellular turgor and storage [306].

Plant aquaporin regulation in response to salt stress involves various mechanisms. Generally, the expression of genes encoding PIPs and TIPs is downregulated in roots following salinity exposure, serving as an osmotic protective response to prevent water loss [307,308,309,310,311]. However, some isoforms have been found to be upregulated or not significantly altered under salt stress, depending on NaCl concentration or plant tissue type [312,313]. Increased expression or stabilization of specific PIP and TIP isoforms may represent an adaptive evolutionary mechanism to saline environments, helping to maintain osmotic homeostasis by controlling water uptake and Na^+^ exchange to compensate for reduced apoplastic pathway functionality and enhance extracellular osmolarity potential [311,314]. Nanopriming with Ag NPs increased expression of aquaporin genes PIP1;1 and PIP2;1, promoting seed water uptake [183]. A previous study also demonstrated that incorporation of CeO_2_ NPs into seed organs (seed coat, cotyledons, and radicle) facilitates rapid seed water absorption [257]. In onions, NaCl concentrations of 25 to 100 mM differentially affected the expression of PIP2, PIP1, and TIP2 aquaporin genes, with significant growth reductions in leaves, roots, and bulbs observed at 50 mM NaCl [315]. Decreases in Mn, Zn, and B content at this concentration were linked to aquaporin expression, suggesting two distinct phases of salinity response based on NaCl levels, with activation of PIP2 at 75 mM being crucial for Zn uptake under high salinity [315].

CeO_2_ NPs elevated the relative expression levels of α-amylase genes (AMY1 and AMY2) and enhanced the expression of *SARD1* (salicylic acid-related disease resistance gene 1) and *PAL* (phenylalanine ammonia-lyase) genes, modulating salicylic acid (SA) biosynthesis in both roots and shoots of rapeseed seedlings and thereby decreasing oxidative damage and increasing salt tolerance [258]. ZnO NPs and SeNPs decreased the expression levels of ABA-related genes (*BnCYP707A1*, *BnCYP707A3*, and *BnCYP707A4*) while increasing GA-related gene expression (*BnGA20ox*, *BnGA3ox*, and *BnCPS*). These findings suggest that the improved salinity tolerance of rapeseed induced by nanopriming with ZnO NPs and SeNPs was correlated with a reduction in ABA levels and an elevation in GA levels [256]. Several POD-related genes (*GhD09G1420*, *GhA03G2152*, *GhD08G2330*, *GhA03G1517*, *Gh09G1415*, *GhD11G2183*, and *GhA10G2288*) were upregulated in CeO_2_ NP-primed cotton plants under salt stress compared to controls [316]. Salinity hinders Mn absorption, impairing Mn-SOD activity and negatively affecting cell division and reproduction [317]. MnNP nanopriming increased SOD enzyme levels by upregulating the *Mn-SOD* gene, thereby preventing ROS damage and phytotoxic effects in pepper [318]. Similarly, MnO NP nanopriming enhanced antioxidant profiles and promoted seedling growth in watermelon [319]. AgNP seed treatment upregulated the *SOS2* gene and conferred increased salt tolerance in tomato [320].

Nano-modified biochar modulated the expression of salt-specific genes in rice plants [321]. Downregulation of *OsSOD4* and *OsMPK6* in the salt-sensitive genotype JL534 suggested that nano-modified biochar effectively alleviates oxidative stress and modulates mitogen-activated protein kinase (MAPK) signaling pathway responses toward a more regulated state [322,323]. Downregulation of *OsERF1* (ethylene response factor 1) in the salt-tolerant genotype XL900 indicated efficient stress mitigation, implying reduced reliance on the ethylene-promoted signaling cascade under stress [324,325]. This coordinated downregulation reflects optimized stress signaling and resource allocation supporting growth and survival under salinity [326]. ZnO-BC20 residues also regulated the expression of antioxidant genes *OsSOD4* and *OsCATA*, as well as salt-tolerance genes *OsWRKY42*, *OsMPK6*, and *OsGAI* [278]. Upregulation of *OsAPX1* and *OsSOD2* in SBC-treated rice under salinity corresponded with increased antioxidant enzyme activity and decreased oxidative damage, suggesting strengthened transcriptional activation of the ascorbate-glutathione cycle for ROS scavenging [299]. A comprehensive overview of NP and NBC types, concentrations, plant species, salinity levels, and their reported germination and biochemical/molecular effects is provided in Table 2.

## 8. Discussion

The findings synthesized in this review highlight the considerable potential of NPs and NBC as tools for improving seed germination and early seedling establishment under salinity stress. However, a critical examination of the available evidence reveals several important limitations and inconsistencies that warrant careful consideration.

A major challenge in comparing studies across the literature is the substantial variability in experimental conditions, including nanoparticle type, size, concentration, application method, plant species, genotype, and salinity intensity. For instance, while TiO_2_ NPs improved GP% and GI in wheat under moderate salinity [254], and ZnO NPs enhanced GR in rapeseed [256], the magnitude of these effects varied considerably across genotypes and salinity levels, underscoring that no universal application protocol currently exists. Similarly, NBC effects on germination indices were concentration-dependent, with 5% NBC showing significant improvements in wheat [35], while lower concentrations produced less consistent results. This dose-dependency, combined with species-specific responses, complicates the extrapolation of findings across crops and environments. A critical question emerging from this synthesis concerns the mechanistic complementarity of NPs and NBC. While both improve germination under salinity, their primary modes of action appear distinct: NPs act predominantly through direct cellular signaling—modulating aquaporin expression, ROS scavenging, and hormonal balance at the seed level—whereas NBC exerts effects largely through soil-mediated pathways, including improved water retention, ion adsorption, and nutrient bioavailability [28,293]. This distinction has important practical implications: NPs appear more effective as seed priming agents, while NBC may be better suited as a soil amendment for sustained salinity management. However, the extent to which these mechanisms overlap—particularly regarding ROS homeostasis and ABA/GA signaling—remains unresolved, and studies combining both approaches are scarce. Future research should explicitly test whether NP + NBC combinations produce additive, synergistic, or antagonistic effects on germination under salinity stress.

Beyond variability in experimental conditions, a fundamental comparability problem exists across studies: germination indices are not uniformly defined or calculated. For instance, germination rate (GR) is computed differently across studies—some use the reciprocal of MGT, others use the number of seeds germinated per day—leading to values that cannot be directly compared [15,16]. Similarly, salinity levels are expressed in different units (mM NaCl, g L^−1^, dS m^−1^) across the reviewed literature, complicating dose response comparisons. The absence of standardized reporting protocols for nanopriming experiments—including nanoparticle characterization, priming duration, substrate type, and germination scoring criteria—represents a systemic barrier to meta-analytic synthesis in this field. Adoption of minimum reporting standards, analogous to those established for nanotoxicology [28], would substantially improve the interpretability and reproducibility of future nanopriming studies.

At the molecular level, while NPs have been shown to upregulate aquaporin isoforms such as PIP1;1 and PIP2;1 [183] and modulate ABA/GA-related gene expression [316], the precise signaling cascades linking NP application to these transcriptional changes remain incompletely understood. Similarly, the mechanisms by which NBC modulates salt-specific gene expression—including *OsWRKY42*, *OsMPK6*, and *OsGAI* [278] require further mechanistic clarification, particularly regarding the role of NBC-derived organic compounds in ABA signaling pathway modulation [303].

A mechanistically underexplored aspect of NP-mediated germination improvement concerns seed coat permeability and nanoparticle uptake routes. Studies with CeO_2_ NPs demonstrated size-dependent distribution across seed coat, cotyledons, and radicle during imbibition [257], while Ag NPs promoted aquaporin-mediated water uptake [183]. However, seed coat composition varies substantially across species and genotypes, influencing both the rate of NP penetration and the downstream physiological responses. This variability may partly explain the species- and cultivar-specific effects documented across studies, and represents a promising target for mechanistic investigation—particularly regarding the relationship between seed coat anatomy, imbibition kinetics, and NP bioavailability during Phase I germination.

Another critical consideration is the potential phytotoxicity of NPs at high concentrations, which has been documented in several studies and may counteract the beneficial effects observed at optimal doses [255,297]. Furthermore, the majority of studies reviewed here were conducted under controlled laboratory or greenhouse conditions, limiting the direct applicability of findings to field-scale saline agriculture. Long-term soil–plant interactions, nanoparticle persistence in the soil, and potential ecotoxicological effects remain insufficiently characterized [28].

The agronomic promise of NPs must be weighed against unresolved safety and regulatory concerns. While optimal concentrations of ZnO NPs, TiO_2_ NPs, and nanoceria improve germination and stress tolerance, phytotoxic effects have been documented at higher concentrations across multiple species [255,297]. Beyond phytotoxicity, the persistence of engineered nanomaterials in soil, their potential accumulation in food chains, and their ecotoxicological effects on soil microbiota and non-target organisms remain poorly characterized [28]. Regulatory frameworks for nanomaterial use in agriculture are still developing in most jurisdictions, and no crop-specific maximum residue limits for nanoparticles currently exist. These gaps represent not only scientific uncertainties but also barriers to commercial adoption and should be addressed as a priority in future field-scale research programs.

Several mechanistically important questions remain unaddressed in the current literature. First, the majority of studies reviewed focused on a limited set of crops—predominantly wheat, rapeseed, and rice—leaving major staple crops such as sorghum, maize under combined stresses, and legumes underrepresented. Second, while this review focused on seed germination as a discrete stage, the carry-over effects of seed nanopriming on subsequent vegetative growth, reproductive performance, and yield under sustained field salinity have been evaluated in only a small number of studies [272,273,293]. Third, the interaction between NPs/NBC and the seed microbiome—which plays an increasingly recognized role in stress priming and germination vigor—has not been systematically investigated. Addressing these gaps would substantially advance the translational potential of nanopriming technologies for saline agriculture.

Environmental and toxicity assessments of biological systems are significantly influenced by the physicochemical properties of these engineered nanomaterials. Thorough studies on these properties are essential for accurate toxicological assessment of NPs [329]. This section explains that the effect of metal-based NPs on seed germination is concentration-dependent and varies significantly by plant species and NP type. High concentrations of metal NPs often suppress germination. Application of Zn NPs at 2000 mg L^−1^ concentration can inhibit germination in crops such as ryegrass and maize due to the presence of dissolved metal cations [330]. Fe NPs (zero-valent iron NPs; 1000 and 2000 mg L^−1^.) and Ag NPs (100 mg L^−1^) are particularly toxic at high levels, producing ROS that cause oxidative stress, damage cell components, suppress germination and inhibit root growth [331]. NPs have both positive and negative effects on seed germination. For example, Fezzi et al. reported that wheat germination rate and biomass increased with low concentrations of (<10 mg L^−1^) TiO_2_ NPs [332]. Generally, NPs have a concentration-dependent effect on seed germination; low concentrations are generally neutral or positive, and most studies of NPs, including ZnO NPs, show that high concentrations negatively affect seed germination and even plant growth and development [333,334,335,336,337].

However, there are limitations and potential risks associated with producing and using nanobiochar for environmental remediation. Therefore, large-scale field applications are recommended [338]. However, it is crucial to assess the long-term impacts on soil health and aquatic ecosystems and to determine specific toxicity thresholds for nanobiochar [339]. To avoid accidental environmental harm, regulations and guidelines should be developed for the safe and sustainable use of nanobiochar [340]. In agriculture, its strong adsorption capacity can cause nutrient-binding issues, potentially resulting in soil degradation and ecological crises [341].

Finally, while this review focused specifically on seed germination as a distinct developmental stage, the transition from germination to seedling establishment and subsequent vegetative growth under sustained salinity stress represents a continuum that has not been fully addressed in the NP and NBC literature. Future research should therefore prioritize field-scale validation with both prospective positive- and negative-effect-based studies, multi-generational assessments of NPs’ and NBC’s effects, and the development of standardized protocols for nanopriming across diverse crop species and salinity regimes.

## 9. Conclusions

Soil salinity is a major abiotic stress that severely affects seed germination and early seedling establishment through osmotic stress, ionic toxicity, oxidative damage, and hormonal imbalance, ultimately impairing water uptake, ion homeostasis, hydrolytic enzyme activity, and antioxidant defense systems. This review demonstrates that NPs and NBC can alleviate salinity-induced constraints on seed germination through multiple morphophysiological, biochemical, and molecular mechanisms. Their application improved all key germination indices—GP%, GR, GI, MDG, and GE—while reducing MGT under saline conditions, with beneficial effects linked to enhanced water uptake, membrane stability, ion regulation, and α-amylase stimulation. At the biochemical level, NPs and NBC reduce oxidative stress markers such as MDA and H_2_O_2_ while enhancing antioxidant enzyme activities (SOD, CAT, POD, APX) and promoting the accumulation of osmoprotective compounds including proline, soluble proteins, and soluble sugars. At the molecular level, NPs and NBC regulate aquaporin gene families, α-amylase-related genes, ABA- and GA-related biosynthetic pathways, SOS signaling, MAPK pathways, and stress-responsive transcription factors, collectively supporting improved salinity tolerance during seed germination and early seedling development.

Critically, this review identifies several gaps that must be addressed before NPs and NBC can be translated into practical saline agriculture tools. The mechanistic complementarity between NPs—which act primarily through direct seed-level signaling—and NBC—which operates largely through soil-mediated pathways—remains underexplored, and their combined application has not been systematically evaluated. Furthermore, the lack of standardized reporting protocols for nanopriming experiments, the underrepresentation of key staple crops, and unresolved regulatory and ecotoxicological concerns represent barriers to field-scale adoption. Addressing these challenges through interdisciplinary research integrating agronomy, nanotoxicology, and molecular plant biology will be essential for realizing the full potential of NPs and NBC in sustainable saline agriculture.

## Figures and Tables

**Table 1:** Effect of salinity stress on key morpho-physiological, biochemical, and molecular traits of seed germination.

Traits	Response Mechanism	Effect on Seed Germination	References
Morpho-Physiological	Osmotic stress	Inhibits imbibition, reducing water uptake through the seed coat	[56,57,59,62,135,186,187,188,189,190,191,192]
	Ionic stress	Increased Na^+^ and Cl^−^ uptake disrupts ion homeostasis and reduces K^+^ levels, leading to nutrient deficiencies	[65,104,105,106,107]
	Hormonal imbalance (↑ ABA, ↓ GA)	Osmotic and ionic stresses increase ABA concentration and decrease GA concentration, promoting seed dormancy	[96,97,98,99,100,101,102]
	Reduced seedling growth traits	↓ GP%, GR, GI, MDG, GE, SVI, shoot length, root length, SDW, STI, FG%, Vc, IV, WC, SL, RL, SFW; ↑ MGT, SI	[18,58,168,193,194,195,196,197,198,199,200,201,202,203]
Biochemical	ROS overproduction (salinity-induced oxidative stress)	↑ ROS (O_2_^−^·, H_2_O_2_, ·OH); ↑ MDA; membrane lipid peroxidation; oxidative damage to lipids, proteins, and nucleic acids	[1,141,204,205,206,207,208]
	Antioxidant defense response (plant compensatory response)	↑ SOD, CAT, APX, GRase; ↑ GSH (glutathione), compatible solutes, phenolics, α-tocopherol (Vitamin E), carotenoids, flavonoids, and proline	[1,204,205,206,207,208]
	Metabolic enzyme	↓ α-amylase enzyme activity	[72,125,131,206,207,209]
Molecular	Signaling and gene expression pathways	Stress signaling transduction (e.g., ABA, GA, ethylene, Ca^2+^, ROS, MAPK, ER stress); ↑ ion transporters, osmolyte biosynthesis genes, antioxidant enzyme genes; ↓ growth-related genes	[103,121,210,211,212,213,214,215]

Note: ↑ increase; ↓ decrease. Abbreviations: germination capacity (GC), final germination percentage (FG%), velocity coefficient (Vc), stress index (SI), salt tolerance index (STI), seedling vigor index (SVI), initial vigor (IV), seedling length (SL, mm), radicle length (RL, mm), seedling fresh weight (SFW), seedling dry weight (SDW), water content (WC), germination percentage (GP%), germination rate (GR), germination index (GI), mean daily germination (MDG), germination energy (GE), mean germination time (MGT), superoxide dismutase (SOD), catalase (CAT), ascorbate peroxidase (APX), glutathione reductase (GRase), glutathione (GSH), peroxidase (POD), abscisic acid (ABA), gibberellin (GA), mitogen-activated protein kinase (MAPK), endoplasmic reticulum (ER), reactive oxygen species (ROS).

**Table 2:** Summary of nanoparticle (NP) and nanobiochar (NBC) types, concentrations, plant species, salinity levels, and key germination and biochemical/molecular effects reported in the literature.

NP/NBC Type	Concentration	Plant Species	Salinity Level	Key Germination Effect	Biochemical/Molecular Effect	Ref.
**Nanoparticles (NPs)**						
TiO_2_ NPs	50 mg L^−1^	Wheat (*Triticum aestivum*)	50–150 mM NaCl	↑ GP% (recovered 59–72% vs. salt-stressed control); ↑ GI (recovered 27–67%)	↑ SOD, CAT; ↓ MDA	[254]
SeNPs + ZnO NPs	SeNPs: 150 µmol L^−1^; ZnO NPs: 25–100 mg L^−1^	Rapeseed (*Brassica napus*)	150 mM NaCl	↑ GR (+42.16–171.4% vs. unprimed)	↓ BnCYP707A1/A3/A4; ↑ BnGA20ox, BnGA3ox, BnCPS	[256]
PNC (nanoceria) (9.2 nm; ζ = −38.7 mV)	Not stated (see ref.)	Rapeseed (*Brassica napus*)	200 mM NaCl	FG%: 84% (PNC+TES) vs. 76% (TES alone)	↑ AMY1, AMY2; ↑ SARD1, PAL; ↑ SA biosynthesis	[257,258]
TiO_2_ NPs	60 mg L^−1^	Maize (*Zea mays*)	Saline soil	↑ GP%, ↑ GE, ↑ SVI	↑ SOD, CAT; ↓ MDA, MEL, MET	[295]
ZnO NPs	750 mg L^−1^ (6 h priming)	Tomato (*Solanum lycopersicum*)	50–100 mM NaCl	↓ MDG loss (12.6–14.1%)	↑ Antioxidant enzyme activity	[261]
ZnO NPs	Varies (see ref.)	Maize (*Zea mays*)	50–100 mM NaCl	↑ GE under saline soil conditions	↑ Osmoprotectant accumulation	[266,327]
nano-Se (87.7 nm)	50–100 mg L^−1^	Wheat (*Triticum aestivum*)	150 mM NaCl	↓ MGT: 4.5 days (75 mg L^−1^) vs. 5.4 days (control)	↑ Antioxidant profiles	[328]
Si NPs (50–100 nm)	Not stated (see ref.)	Wheat (*Triticum aestivum*)	100 mM NaCl	↓ MGT; ↑ germination by breaking dormancy	↑ Water uptake; ↑ seedling growth	[265]
ZnO NPs	200 mg L^−1^	Rice (*Oryza sativa*)	0–3% NaCl	↑ Seedling growth and biomass	↑ Chlorophyll; ↑ photosynthetic capacity	[273]
ZnO NPs	25–100 mg L^−1^	Rapeseed (*Brassica napus*)	150 mM NaCl	↑ Shoot length (+9.60–25.63%); ↑ root length (+41.64–48.17%)	↑ Biomass; ↑ antioxidant defense	[272]
ZnO NPs	Varies (see ref.)	Pea (*Pisum sativum*)	Saline conditions	↑ Salinity tolerance	↑ CAT, POD, PPO enzyme levels	[297]
CNPs	80 mM	Radish (*Raphanus sativus*)	25 mM NaCl	↑ Sprouting; ↑ antioxidant activity	↑ Polyphenols, flavonoids, polyamines, anthocyanins, proline	[296]
CeO_2_ NPs (PNC) (9.2 nm; ζ = −38.7 mV)	Not stated (see ref.)	Rapeseed (*Brassica napus*)	Saline conditions	↑ Seed water absorption (seed coat, cotyledons, radicle)	↑ AMY1, AMY2; ↑ SARD1, PAL; ↑ SA biosynthesis	[257,258]
Ag NPs (6–36 nm)	10–20 mg L^−1^	Rice (*Oryza sativa*); Tomato (*Solanum lycopersicum*)	Saline conditions	↑ Water uptake via aquaporins (PIP1;1, PIP2;1)	↑ SOS2 gene expression; ↑ salt tolerance	[183,320]
MnNPs (Mn_2_O_3_)	0.1–1 mg L^−1^	Pepper (*Capsicum annuum*)	100 mM NaCl	↑ Root elongation; ↑ SOD activity; ↓ ROS damage	↑ Mn-SOD gene expression; ↓ phytotoxicity	[318]
MnO NPs	Varies (see ref.)	Watermelon (*Citrullus lanatus*)	Saline conditions	↑ Seedling growth	↑ Antioxidant profiles	[319]
**Nanobiochar (NBC) and Nano-modified Biochar**						
NBC (nanoscale fraction 10–100 nm)	0–5% (*w*/*w*)	Wheat (*Triticum aestivum*)	80 mM NaCl	↑ GP%, ↑ GI; ↑ radicle and plumule length	↑ Hydrolytic enzyme activity; ↑ N-metabolic enzymes	[35]
ZnOBC-20	BC: 20 t ha^−1^ + ZnO NPs: 20 mg L^−1^	Rice (*Oryza sativa*)	EC ≈ 10.5 dS m^−1^	↑ Shoot and root biomass; ↑ RWC	↑ SOD (10–10.9%), POD (11.6–19.7%), CAT (17.1–9.7%), APX (12.9–6.9%); ↑ K^+^/Na^+^	[293]
ZnBC@2	BC: 20 t ha^−1^ + ZnO NPs: 20 mg L^−1^	Rice (*Oryza sativa*)	EC ≈ 10.5 dS m^−1^	↑ Seedling establishment; ↑ stress tolerance	↑ SOD, POD, CAT, APX; ↑ proline via P5CS; ↑ OsWRKY42, OsGAI	[321]
FeBC@1	BC: 20 t ha^−1^ + Fe_2_O_3_ NPs: 10 mg L^−1^	Rice (*Oryza sativa*)	EC ≈ 10.5 dS m^−1^	↑ Seedling growth	↑ SOD, POD, CAT, APX (less than ZnBC@2)	[321]
SBC (SiO_2_-biochar)	Varies (see ref.)	Rice (*Oryza sativa*)	Saline conditions	↑ Cellular integrity	↓ H_2_O_2_, ↓ MDA; ↑ SOD, CAT, POD; ↑ OsAPX1, OsSOD2	[299]
ZnO-BC20 (residual)	BC: 20 t ha^−1^ + ZnO NPs: 20 mg L^−1^	Rice (*Oryza sativa*)	Saline soil	↑ Plant height; ↑ photosynthetic efficiency	↑ OsSOD4, OsCATA, OsWRKY42, OsMPK6, OsGAI	[293]
PNC (nanoceria); PAC inhibitor control (PNC: 9.2 nm; ζ = −38.7 mV)	PNC: not stated; SA/PAC: see ref.	Rapeseed (*Brassica napus*)	Saline conditions	↑ GP% by 226% (SA+PAC) and 198% (PNC+PAC) vs. PAC-only; PAC alone suppressed germination to 76% under non-saline conditions	↑ SA biosynthesis; ↑ antioxidant defense; ↑ germination uniformity	[258]

Note: ↑ increase; ↓ decrease. Units: Solution-phase concentrations are harmonized to mg L^−1^ (µg mL^−1^, mg L^−1^ and ppm are numerically equivalent for dilute aqueous suspensions); molar concentrations are retained as reported, since conversion depends on the molar mass of the nanomaterial. Soil-amendment doses (% *w*/*w*; t ha^−1^) are physically distinct quantities from suspension concentrations and are therefore not converted. Nanomaterial size and, where reported, zeta potential are given in parentheses in the NP/NBC Type column; these values had previously been placed in the Concentration column in error. Sizes are omitted where the original study does not report particle characterization, which is itself indicative of the lack of standardized reporting discussed in Section 7. “Not stated (see ref.)” = the application concentration is not uniformly reported across the cited studies; readers are directed to the original references for specific values. GP% = germination percentage; GI = germination index; GE = germination energy; GR = germination rate; MDG = mean daily germination; MGT = mean germination time; FG% = final germination percentage; SVI = seedling vigor index; RWC = relative water content; MDA = malondialdehyde; MEL = membrane electrolyte leakage; MET = mean emergence time; SOD = superoxide dismutase; CAT = catalase; APX = ascorbate peroxidase; POD = peroxidase; PPO = polyphenol oxidase; SA = salicylic acid; PAC = salicylic acid biosynthesis inhibitor (used as experimental control to confirm SA-mediated mechanism of PNC action); PNC = polyacrylic acid-coated nanoceria; CNPs = carbon nanoparticles; SBC = SiO_2_-biochar formulation; P5CS = pyrroline-5-carboxylate synthase; NBC = nanobiochar; BC = biochar; EC = electrical conductivity; TES = trisodium EDTA solution (10 mM), used as vehicle/carrier control for PNC priming experiments.

## Data Availability

Data sharing is applicable upon reasonable request from the corresponding author.
